# Pharmacological Inhibition of MALT1 Protease Leads to a Progressive IPEX-Like Pathology

**DOI:** 10.3389/fimmu.2020.00745

**Published:** 2020-04-30

**Authors:** Kea Martin, Ursula Junker, Elaine Tritto, Esther Sutter, Tina Rubic-Schneider, Hannah Morgan, Satoru Niwa, Jianping Li, Achim Schlapbach, Dana Walker, Marc Bigaud, Christian Beerli, Amanda Littlewood-Evans, Bettina Rudolph, Marc Laisney, David Ledieu, Karen Beltz, Jean Quancard, Frédéric Bornancin, Natasa Zamurovic Ribrioux, Thomas Calzascia

**Affiliations:** ^1^Autoimmunity, Transplantation and Inflammation, Novartis Institutes for Biomedical Research, Basel, Switzerland; ^2^Preclinical Safety, Novartis Institutes for Biomedical Research, Basel, Switzerland; ^3^Global Discovery Chemistry, Novartis Institutes for Biomedical Research, Basel, Switzerland; ^4^Preclinical Safety, Novartis Institutes for Biomedical Research, Cambridge, MA, United States; ^5^PK Sciences, Novartis Institutes for Biomedical Research, Basel, Switzerland

**Keywords:** MALT1, regulatory T cells, autoimmune disease, inflammation, toxicology

## Abstract

Genetic disruption or short-term pharmacological inhibition of MALT1 protease is effective in several preclinical models of autoimmunity and B cell malignancies. Despite these protective effects, the severe reduction in regulatory T cells (Tregs) and the associated IPEX-like pathology occurring upon congenital disruption of the MALT1 protease in mice has raised concerns about the long-term safety of MALT1 inhibition. Here we describe the results of a series of toxicology studies in rat and dog species using MLT-943, a novel potent and selective MALT1 protease inhibitor. While MLT-943 effectively prevented T cell-dependent B cell immune responses and reduced joint inflammation in the collagen-induced arthritis rat pharmacology model, in both preclinical species, pharmacological inhibition of MALT1 was associated with a rapid and dose-dependent reduction in Tregs and resulted in the progressive appearance of immune abnormalities and clinical signs of an IPEX-like pathology. At the 13-week time point, rats displayed severe intestinal inflammation associated with mast cell activation, high serum IgE levels, systemic T cell activation and mononuclear cell infiltration in multiple tissues. Importantly, using thymectomized rats we demonstrated that MALT1 protease inhibition affects peripheral Treg frequency independently of effects on thymic Treg output and development. Our data confirm the therapeutic potential of MALT1 protease inhibitors but highlight the safety risks and challenges to consider before potential application of such inhibitors into the clinic.

## Introduction

Mucosa-associated lymphoid tissue lymphoma translocation protein 1 (MALT1) was coined based on the discovery of its translocated gene product in patients with MALT lymphoma (1, 2). Shortly afterward, MALT1 emerged as an essential scaffolding component of CBM complexes, which are formed together with a CARMA/CARD protein and BCL10 (B cell lymphoma/leukemia 10) (3, 4). The CBM complex nucleated by CARMA-1/CARD11 is critical for antigen-receptor dependent regulation of transcriptional responses, in particular those driven by nuclear factor-kappa B (NF-κB) (5). In addition to a well-understood scaffolding function, MALT1, also known as paracaspase-1, displays arginine-directed proteolytic activity (6–8). The discovery of MALT1 paracaspase activity triggered an intense research interest, providing a novel paradigm to dampen pathological hyperactivity of CBM pathways, which MALT1 contributes to by proteolytic cleavage of several key regulators among which A20, CYLD, RelB, and BCL10 (9), be it in lymphoma settings (10, 11) or in autoimmune/auto-inflammatory disorders (12).

Genetic models of MALT1 deficiencies and pharmacological MALT1 protease tool compounds have been described (3, 4, 13–19, 20) and a number of encouraging studies were published. MALT1 deficiency or short-term pharmacological protease inhibition attenuated the onset and progression of experimental autoimmune encephalomyelitis (EAE) (12, 13, 21, 22), reduced immune complex-driven arthritis (20) and was beneficial for the outcome of DSS colitis in mice (23, 24). In addition, MALT1 protease inhibition reduced the tumor burden in human ABC-DLBCL xenograft models (18, 19). Surprisingly though, congenital disruption of MALT1 protease function resulted in a spontaneous and lethal Immunodeficiency-Polyendrocrinopathy and Enteropathy-X-linked (IPEX)-like multi-organ inflammatory pathology in MALT1 protease-deficient (MALT1 PD) animals, caused by an impaired regulatory T cell (Treg) compartment (13–16). The animals displayed lymphadenopathy, elevated serum IgG1 and IgE levels, expansion of effector T cells and lymphocytic infiltrates in various tissues. The exact cause of death associated with these findings has remained unclear but contribution of interferon γ-producing cells in mediating the neuropathology (14), and antibodies driving autoimmune gastritis (15) was proposed. We recently determined that the systemic and lethal disease component is mediated by T lymphocytes, while dysregulated humoral responses at environmental barriers lead to the production of microbiota- and food-reactive IgG1 and IgE antibodies in MALT1 PD mice (25). By contrast, the lack of lymphocyte effector functions in immunodeficient MALT1 KO mice results in only mild clinical symptoms despite a more severe reduction in Treg cells (13–16). Very recently, four novel reports elucidated the role of the CBM complex in Treg function and the maintenance of immune homeostasis (26–29). Genetic deletion of CARD11, BCL10, or MALT1, as well as the genetic inactivation of the MALT1 protease selectively in Foxp3^+^ Tregs was associated with Treg dysfunction *in vivo* and development of an IPEX-like disease (26, 28, 29). In line with this, T cell-restricted inactivation of MALT1 protease is sufficient to cause an IPEX-like pathology similar to the one observed in full-body MALT1 PD animals (27). Of note, using MALT1 PD Treg cells or WT Treg cells treated with a MALT1 inhibitor, various groups suggested that impairment of MALT1 protease function in Tregs leads to defective upregulation of several molecules associated with Treg suppressive activity, such as CTLA-4, IL-10, and TGF-β (26, 27, 29). Overall, these studies indicate that CBM components including MALT1 protease function are critical to maintain the optimal suppressive function and identity of Tregs *in vivo.*

Several human patients harboring somatic or germline mutations impacting the CBM complex have been reported over the last decade (30). In humans, loss of function mutations in *MALT1* generally result in reduced MALT1 protein levels and cause an inborn immunodeficiency that combines increased sensitivity to all types of infections with an IPEX-like syndrome, which is fatal unless treated with hematopoietic stem cell transplantation (31–35). Patients with *MALT1* mutations present with autoimmune enteropathy, dermatitis, and hyper IgE considered to be caused by deficiency in FoxP3^+^ Tregs (31). Thus, clinical manifestations in humans resemble the MALT1 PD mouse pathological symptoms to a certain degree (13–16).

As a result, the therapeutic potential of MALT1 protease inhibition has become questionable (36). It is therefore of utmost importance to ask whether abrogating MALT1 protease function in adult individuals might lead to an autoimmune pathology similar to the congenic human genetic mutations or to the MALT1 PD murine models. Here we used MLT-943, a novel MALT1 protease inhibitor displaying high potency and selectivity both *in vivo* and *in vitro*, to assess the long-term safety of MALT1 protease inhibition in two preclinical species.

## Materials and Methods

### Synthesis and Characterization of MLT-943

The synthesis of MLT-943 was performed as described in WO2015/181747 or in the Supplementary Material. MLT-943 compound characterization: 1H NMR (400 MHz, DMSO-d6), δ: 10.36 (s, 1H), 8.92 (s, 1H), 8.56 (d, *J* = 5.6 Hz, 1H), 8.06 (d, *J* = 2.0 Hz, 1H), 7.61 (dd, *J* = 5.6, 2.0 Hz, 1H), 6.92 (s, 1H), 5.41 (q, *J* = 6.7 Hz, 1H), 3.32 (s, 3H), 1.57 (d, *J* = 6.7 Hz, 3H). 13C NMR (101 MHz, DMSO-d6) δ: 152.79, 150.85, 149.97, 148.03, 147.3 (q), 146.35, 144.45, 138.88, 121.6 (q), 120.03, 114.86, 108.89, 95.47, 72.93, 57.24, 17.40. HR-MS: [M + H]^+^ C_16_H_15_ClF_3_N_6_O_2_ calculated: 415.08916, found: 415.08914.

MLT-943 was administered orally by gavage as a nanosuspension in 2% (w/v) Kollidon VA64 in Purified Water, USP containing 0.1% (w/v) Sodium Lauryl Sulfate, unless specified differently (see rat collagen-induced arthritis).

### *In vitro* Pharmacology Profiling

The IL-2 reporter gene assay in Jurkat T cells, the IL-2 release assay in primary human T cells, the CYLD cleavage assay in human primary T cells, and the IL-2 release assay in human PBMC, were done with MLT-943 here as previously reported for MLT-827 (17). Human whole blood was obtained from healthy volunteers by venipuncture at the Novartis Basle Health care unit. Blood was pooled into two 50 ml Falcon tubes and filtered using a 100 μm (or 70 μm)-cell strainer. Serial dilutions of compounds (10 mM DMSO stock) were diluted again 1/10 in X-VIVO medium (Lonza Biosciences) and 500 μl of whole blood was mixed with 55 μl of X-VIVO/compound mixes and pre-incubated for 1 h at 37°C with shaking (∼600 rpm). After the pre-incubation time 200 μl of the blood/compound mixes in duplicates were transferred to a new flat bottom plates and PMA (final: 100 ng/ml)/anti-CD28 (final: 300 ng/ml) solution was added. Incubation was performed overnight at 37°C + 5% CO2. Blood plates were spun for 10 min at 470 g and 100 μl of the resulting serum was analyzed for IL-2 levels using MesoScale Discovery kits (MSD), according to the manufacturer’s protocol. For delayed measurement, a lid was sealed and the plate was frozen at −80°C. Similar protocols were implemented for PBMC and blood from rat and dog species.

### Kinase/Protease/Off-Target Panels

Biochemical assays to determine the inhibitory potency of MLT-943 for a panel of enzymes were run in 384 well microtiter plates. Serial dilutions of MLT-943 and reference inhibitors, as well as controls for high and low signal were included in each plate. *Z*’ values, calculated from the controls, were required to be above 0.5 (37). Conditions were optimized for each assay and enzyme reactions were linear for the indicated reaction time at the applied enzyme and substrate concentrations.

Kinase reactions were started by mixing test compound (0.05 μl) with substrate peptide/ATP solution (4.5 μl) and kinase solution (4.5 μl). After a reaction time of 1 h at 30°C, the enzymatic reaction was stopped by adding stop/run buffer, containing 10 mM EDTA. Phosphorylated and unphosphorylated peptides were separated using microfluidic mobility shift technology (Caliper LC3000), allowing the determination of kinase activities.

For protease reactions, test compound (0.25 μl) with protease solution (12.5 μl) were mixed and pre-incubated for 70 min at RT, before starting the enzymatic reaction by addition of substrate peptide solution (12.5 μl). After a reaction time of 1 h at RT, a fluorescence signal was measured (fluorescence intensity or lifetime (38) depending on the enzyme/substrate combination), allowing the determination of the amount of formed product and hence protease activities.

With the resulting values, dose–response curves and corresponding IC50 values were fitted using a sigmoidal four-parameter fit.

### Collagen-Induced Arthritis (CIA) in Rats

Freund’s incomplete adjuvant (IFA, Difco, Detroit, United States) was mixed with porcine collagen type II (Chondrex, Redmond, United States) using a polytron on ice. The final solution consisted of 200 μg of collagen in 200 μl of IFA. Two hundred ml of this was injected intradermally (i.d.) into the base of the tail of an isofluorane narcotized female Lewis rat. After 7 days, the isofluorane narcotized animal was boosted i.d. with a fresh batch of the immunization solution in an adjacent site at the base of the tail (100 μg in 100 μl). Paw swelling and body weight were taken three times per week as described below.

For studies involving prophylactic treatment, MLT-943 was suspended in 40% MEPC5 vehicle and was administered p.o. at a dose of 10 mg/kg once per day, starting at day 0 and continuing until day 20. There were 10 animals per group for the compound treated groups and *n* = 9 rats for the vehicle group. On day 21, 21 h after last dose, animals were sacrificed and 100 μl of serum was taken for anti-collagen antibody titer. Hind paws were taken for histological analysis.

For studies involving therapeutic treatment, MLT-943 was suspended in MC 0.5%/Tween 80 0.5% in water and was administered p.o. at a dose of 3 or 10 mg/kg once per day, starting at day 15, 8 days after boost and continuing until day 23. There were five animals per group. On day 24, animals were sacrificed without further dosing (24 h post last dose) and hind paws were taken for histology.

All animal studies were performed according to current Swiss animal protection laws, issued by the Cantonal Veterinary Office Basel-Stadt, Switzerland. Body weight of the animals was assessed regularly throughout the study. Mean values of groups were calculated. Scoring of the paw swelling was done on an arbitrary scale of 0–12 per rat, evaluating each hind paw in the front with 0–3 and in the ankle with 0–3, thus obtaining a maximal score of 6 per paw. The paw scores were summed up to obtain a score for each individual animal. The individual sum scores of all the animals were averaged and SEMs were calculated. The scoring system used was: 0 = no detectable sign of inflammation; 1, light swollen region; 2, more obviously swollen region; 3, ankylosis or severely swollen region.

### Anti-collagen Type II ELISA

Enzyme linked immunosorbent assay (ELISA) for rat IgG anti rat-Collagen Type II (Chondrex, Catalog Number 1024) was performed on serum from rats according to manufacturer’s instructions. Serum obtained at the end of the study, was diluted 1:10000 and 1: 100000.

### Histopathology Rat CIA

Hind paws samples were fixed in 10% buffered formalin for 48 h, decalcified over 16 days in Immunocal (ref 1440, Decal Chemical Corp, Tallman, NY 10982-0916 United States) changed every 2nd day, processed and embedded in paraffin. Three-μm thick sections were stained with Giemsa and Safranin O (prophylactic setting) or hematoxylin and eosin (therapeutic setting).

Histopathological changes were blindly scored on a scale of 0 (normal) to 3 (severe changes). Following parameters were assessed: inflammatory cell infiltrates, joint damage and proteoglycan loss (44).

### SRBC Immunization, PK Sampling and ELISA

Naive OFA rats (RA238; female) were injected *i.v.* with 1 × 10^8^ sheep erythrocytes for immunization on day 0. MLT-943 (BID and QD) or vehicle treatment was initiated 2 days prior to SRBC-immunization. Blood was harvested on day 3, 3 h post dosing for peak PK levels (50 μl in EDTA-coated tubes) and for the whole blood (WB) assay (200–500 μl in heparin-coated tubes). Terminal bleeding was performed on day 4 for PK (12 h-trough levels), WB assay, and serum collection to assess anti-SRBC IgM by ELISA (Life Diagnostics).

### *In vivo* Treatment With MLT-943 and Mepazine in Mice

Eight-week-old C57BL/6 mice (Charles River France) were treated from day 0 to day 12, either via p.o. (MLT-943) or i.p. (mepazine) dosing. Mepazine acetate (Chembridge, San Diego, CA, United States) was solubilized and dosed in PEG200/water (30/70 v/v). Dosing volume was 10 ml/kg bodyweight for both MLT-943 and mepazine. Some animals were euthanized at the end of the treatment period for analysis of lymphoid organs, while other animals were kept until day 19 to analyze the recovery of Treg cells after cessation of the treatment. All mice were bled on day -1 for assessment of baseline Treg levels and activation markers on Treg cells in blood. Blood samples were collected along the treatment and recovery period to monitor changes in Tregs levels over time. Treg frequencies in blood and spleen as well as the expression of CTLA-4, TNFR2, KLRG1, and CD25 on Tregs was assessed by flow cytometry (see below). All animal studies were performed in accordance with the animal experimentation laws and guidelines laid down by the Swiss Federal and Cantonal Authorities.

### Flow Cytometry

Immunophenotyping of lymphocyte subsets in peripheral blood in rat and dog toxicology studies was conducted at pre-test (twice), week 5 and at the end of the main and recovery period. Splenocytes were analyzed at scheduled necropsies. Similarly, mouse blood cells were harvested by tail bleeding and splenocytes were analyzed upon termination of the animals. Generally, blood was harvested in EDTA-coated tubes and red blood cells (RBCs) were lysed using ACK hypotonic solution. Cell suspensions from spleen and LN were prepared by passing tissues through a 70-μm sieve followed by RBC lysis using ACK hypotonic solution. Cells were washed once in FACS-buffer (PBS containing 2% FCS, 0.05% NaN_3_), blocked with Fc Block (rat: anti-CD32, clone D34-485, BD Biosciences; mouse: anti-CD16/32, clone 2.4G2, BD Biosciences), and stained for 30 min at 4°C with the indicated combination of fluorochrome-conjugated Abs. Intracellular stainings were performed using the FoxP3 Fix/Perm Buffer Set (Biolegend). Cells were acquired using a BD FACSFortessa flow cytometer and data were analyzed using the FlowJo software. For analysis of rat samples, all populations were pre-gated on singlets and live lymphocytes based on their SSC vs. FSC profiles and using appropriate live/dead stains. From live lymphocytes, cells were further gated on CD3, CD4 or CD8 for T cells as indicated on graphs, followed by assessment of the specified activation markers. In case of CD4^+^ T cells, populations were further divided based on FoxP3 and CD25. For analysis of mouse samples, populations were pre-gated on singlets and live lymphocytes based on their SSC vs. FSC profiles and using appropriate live/dead stains. From live lymphocytes, cells were further gated on CD4, and within CD4 + T cells on Foxp3 + cells (clone: FJK-16S, eBioscience) to identify Tregs. Expression levels of CTLA-4 (clone: UC10-4B9, BioLegend), TNFR2 (CD120b, clone: TR75-89, BioLegend), KLRG1 (clone: 2F1/KLRG1, BioLegend) and CD25 (clone: PC61, BD) were assessed on Foxp3 + Treg cells as indicated on graphs. CTLA-4 expression was assessed intra- and extracellularly.

For detection of intracellular IFNγ in rat samples by flow cytometry, cells were isolated from spleen, mandibular LN, or blood and stimulated with 10 ng/mL PMA and 1 μM ionomycin in presence of 10 μg/mL BrefeldinA (all Sigma Aldrich) for 4 h at 37°C prior to FACS staining.

Absolute counts of each population in peripheral blood (10^9^/L) were calculated from the absolute lymphocyte count (LYMA) reported from the ADVIA 2021i (Siemens Medical Solutions Diagnostics).

### Rat Thymectomy Study

During the surgical procedure, animals (10-week-old female and male Wistar rats, Charles River Germany) were kept under continuous 2% isoflurane anesthesia and the skin was cleaned and disinfected by Betaseptic^®^. A midline incision was made from the base of the neck posteriorly over the thorax (2–2.5 cm). The thorax was opened by cutting through the sternal manubrium and pectoral muscles, the connective tissue was torn/snipped and the thymus was pulled out with the aid of a slim cotton-tipped applicator and artery forceps. To prevent pneumothorax, the chest was gently compressed between thumb and forefingers before the cavity was closed and sutured. In sham-operated animals, the same procedure was applied without removal of the thymus. Animals were allowed to recover for at least 2 weeks after thymectomy (or sham-operation) before start of compound treatment.

MLT-943 was applied at 20 mg/kg/day p.o., from the 2nd week post thymectomy onward and for 9 weeks. Leukocyte populations in EDTA-anticoagulated blood were measured using an ADVIA 2021i (Siemens Medical Solutions Diagnostics).

### Toxicology Studies Design

All toxicology studies were conducted based on requirements of the Good Laboratory Practice (GLP) Regulations, with the exception of the immunophenotyping and exploratory analyses.

Four and 13-week rat studies: All animals received humane care according to local welfare laws and guidelines, and the studies were conducted in compliance with US and EU animal health regulations. MLT-943 was administered orally by gavage (formulation as above) to four groups of Wistar Hannover rats (10 or 16/sex/group; Charles River Laboratories, Raleigh, NC, United States, approximately 10-weeks old at start of dosing) at doses of 0, 5, 20 or 80 mg/kg/day for at least 4 or 13 weeks, respectively. The dosing volume for all dose groups was 5 mL/kg, animals were dosed once daily. At the end of the dosing period, six rats/sex from the control and high-dose groups were placed on recovery for 4 or 8 weeks, respectively. Animals placed on study were selected based on available results from the pretest examinations (e.g., body weights, clinical observations, eye examinations) and randomly assigned to dose groups based on body weight and sex. Monitoring for clinical signs occurred at least twice daily, body weight and food consumption was measured at least once weekly. Evaluations were performed based on clinical observations, body weights, food consumption, ophthalmic observations, ECGs, clinical pathology, macroscopic observations, histopathology and toxicokinetics. Additionally, immunohistochemistry, immunophenotyping, gene expression analysis and circulatory biomarker analysis were used as supportive data.

Four and 13-week dog toxicity studies: Beagle dogs were obtained from Marshall BioResources, North Rose, New York, United States. All animals received humane care according to local welfare laws and guidelines, and the studies were conducted in compliance with US and EU animal health regulations. At the initiation of dosing, the dogs were approximately 9 months of age. Animals placed on study were selected and randomized based on available results from pretest evaluations as exemplified in rat study. Littermates were not assigned to the same group. Monitoring for clinical signs occurred at least twice daily, body weight and food consumption were measured at least once weekly. Evaluations were performed based on clinical observations as exemplified in rat study. MLT-943 (formulation as above) was administered orally by gavage to 4 groups of beagle dogs (3 or 5/sex/group) at doses of 0, 0.5, 2, 5 mg/kg/day for at least 4 weeks, or at doses of 0, 2, and 5 mg/kg/day for at least 13 weeks and at 10 mg/kg/day (days 1–50) reduced to 7 mg/kg/day (from day 55 onward). The dosing volume for all dose groups was 5 mL/kg, animals were dosed once daily. At the end of the dosing period, 2 animals/sex from the control and high-dose groups were placed on recovery for at least 6 or 8 weeks, respectively.

### Toxicokinetics

Four and 13-week rat toxicity studies: Blood was obtained from all surviving non-recovery study animals on study days 1–2, study days 28–29 (4-week toxicity study) and on study days 72–73 (13-week toxicity study). Up to two animals/sex/group were bled at approximately 1, 2, 4, 8, and 24 h post-dose during the dosing period. Approximately 0.5 mL of whole blood was collected from the sublingual vein of isoflurane/O_2_-anesthetized animals into tubes containing EDTA and placed on wet ice. A toxicokinetic blood sample was also obtained from the animals euthanized moribund during the study.

Four and 13-week dog toxicity studies: Blood was obtained from all surviving animals on study days 1–2, study days 24–25 (4-week-toxicity study) and on study days 90–91 (13-week toxicity study). Animals were bled at approximately 1, 2, 4, 7 and 24 h post-dose. Approximately 2 mL whole blood was collected into vacutainers with EDTA tube and placed on wet ice.

All blood samples were centrifuged to obtain plasma samples that were stored frozen at −65°C or below. Concentrations of MLT-943 in plasma were measured using a validated LC-MS/MS method. Toxicokinetic parameters included T_*max*_, C_*max*_, C_*max*_/dose, AUC_0__–__24__*h*_, AUC_0__–__24__*h*_/dose were calculated using Watson LIMS software. C_*trough*_ was calculated as deemed appropriate.

### Clinical Pathology

Hematology and clinical biochemistry samples were collected from surviving animals in treatment weeks 5 and 13 and recovery week 8. Urine was collected on surviving animals in treatment weeks 5, 10, and 13 and recovery week 8. Coagulation samples were collected at scheduled necropsies (treatment week 14 and recovery week 9). Hematology was assessed using the ADVIA 2120 Automated Hematology Analyzer (Siemens Medical Solutions Diagnostics). Coagulation parameters were assessed using ACL TOP 500 CTS Hemostasis System (Instrumentation Laboratory) and clinical chemistry parameters using the Cobas c501 System (Roche Diagnostics) with appropriate kits. Urinalysis was done with the Clinitek Atlas Automated Urine Chemistry Analyzer (Siemens Medical Solutions Diagnostics).

### Histopathology

#### Rat

Representative samples of tissues of all animals were collected at necropsy. Fixation and storage of specimens were in 10% neutral-buffered formalin, except for the initial fixation of the testes, eyes and harderian glands (Davidson’s). Tissues were processed to hematoxylin and eosin-stained sections.

#### Dog

For histopathology, tissue samples from selected organs of all animals were fixated in 10% neutral-buffered formalin, except for the initial fixation of the testes and eyes (Davidson’s), and processed to hematoxylin and eosin–stained sections. To assess the nature of mononuclear cell infiltrates in the liver and stomach, T and B cells were immunohistochemically labeled using an anti-CD3 antibody (rabbit polyclonal anti-human CD3 – Dako, Carpinteria, CA, United States, catalog number A 0452) or an anti-CD20 antibody (rabbit polyclonal anti-human CD20 – ThermoFisher Scientific, Grand Island, NY, United States, catalog number PA5-16701). All steps of the immunohistochemical staining were performed on-board a Leica Bond RX autostainer using Leica BOND Polymer Refine Detection kit (Leica, Buffalo Grove, IL, United States).

### Gene Expression in Jejunum

Rat jejunum tissues, harvested at scheduled necropsy (13-week toxicity study in rats) for genomics assessment, were immediately snap-frozen in liquid nitrogen and stored at approximately −80°C until processing. Total RNA was obtained by Trizol/RNeasy (Qiagen) extraction method with a DNase treatment step to remove DNA contamination. Total RNA was quantified by the absorbance at 260 nm, and the quality determined using an Agilent 2100 Bioanalyzer (Agilent Technologies). GeneChip microarray experiments were conducted using Rattus norvegicus Rat230_2 Arrays (Affymetrix, Inc., Santa Clara, California, United States). Approximately 100 ng of total RNA was used to obtain mRNA using the 3′ IVT express kit (Affymetrix, Inc., Santa Clara, United States). For each sample, 10 μg of biotin-labeled cRNA was hybridized on an array for approximately 16 h at 45°C. The array was washed and stained on Affymetrix Fluidics Workstation 450 and scanned on Affymetrix Scanner 3000 according to the manufacturer’s technical manual. The scanned image was converted into numerical values of the signal intensity (signal) and the Affymetrix MAS 5.0 software was used to scale the average intensity of each chip to a target intensity of 150. Fold change values were calculated by comparing the raw expression values for samples from treated animals to controls using internal software, with raw values <0.01 set equal to 0.01 prior to fold change calculations. Up- or down-regulated gene lists generally included only those probe sets where there was a ≥1.3-fold change in expression for treated versus controls. Because of strong inter-animal biological variability, no statistical cut-off was applied in this particular study. Gene signatures, including a set of genes with highly correlated expression profiles belonging to the same molecular pathway or cell type, for mast cells, were initially generated from genes having more than 0.75 Pearson’s correlation coefficient using a complete linkage method. The signatures were then manually curated to exclude genes not considered biologically relevant and to include genes with biological significance even if at lower correlation coefficients. Scores were calculated as geometric mean of fold changes of all genes of the signature.

### Statistics

Means and standard deviations were calculated and an Analysis of Variance (ANOVA) performed on time-point specific data sets (e.g., body weights, food consumption, hematology, coagulation, clinical chemistry, quantitative urinalysis and immunophenotyping). The ANOVA was followed by a Bartlett’s test for homogeneity of variances. When the variances were homogeneous, Dunnett’s *t*-test was employed to determine the statistical significance between control and treated groups. When the variances were not homogeneous, a modified *t*-test was used to determine which groups were statistically different from the controls. Dunnett’s Plasma concentration-time data were analyzed using suitable validated methods.

## Results

### Rat and Dog Are Pharmacologically Relevant Species for Investigating the Safety of MALT1 Protease Inhibition

MLT-943 is a new pyrazolopyrimidine derivative (Table 1), which we characterized as a potent MALT1 inhibitor displaying good pharmacokinetics properties (Supplementary Tables S2–S5) and high selectivity against a range of proteases and other selected off-target panels (Supplementary Table S1). To assess the suitability of MLT-943 for long-term preclinical studies, we measured its ability to inhibit MALT1 paracaspase activity of the human, rat, and dog enzymes. We used T-cell-based assays and measured IL-2 production, which is a major outcome of antigen receptor signaling and is very sensitive to MALT1 proteolytic function (17). Stimulated IL-2 secretion in PBMC or in whole blood was inhibited by MLT-943 with a similar IC_50_ across species (0.07–0.09 μM in PBMC, 0.6–0.8 μM in whole blood), thereby establishing the pharmacological relevance of rat and dog species for conducting safety investigations (Table 1).

**TABLE 1:** MLT-943 pharmacology in human cells and cross-species comparison.

MLT-943 chemical structure			

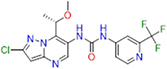			

MLT-943 pharmacology testing	Stimulus	IC50 (μM)	Extent of inhibition (%)
**Human**			
IL-2 reporter gene assay (Jurkat T cells)	PMA/CD28	0.04 ± 0.02	>90
IL-2 release (primary T cells)	CD3/CD28	0.010 ± 0.002	>90
CYLD cleavage (primary T cells)	PMA/ionomycin	0.06 ± 0.007	>90
IL-2 release (PBMC)	PMA/ionomycin	0.074 (*N* = 1)	>80
IL-2 release (50% whole blood) RAT	PMA/CD28	0.80 ± 0.20	>90
**Rat**			
IL-2 release (50% whole blood)	PMA/CD28	0.58 + 0.15	>90
**Dog**			
IL-2 release (PBMC)	PMA/ionomycin	0.092 (*N* = 1)	>80
IL-2 release (50% whole blood)	PMA/CD28	0.58 + 0.04	>90

### Effective MALT1 Protease Inhibition Leads to a Reduction in Treg Frequency

All reported congenital MALT1 PD mouse lines display disruption of immune homeostasis related to reduced Treg proportions and undergo a spontaneous and progressive multi-organ inflammatory pathology (13–16). While congenital MALT1 protease deficiency results in reduced thymic Treg development and reduced peripheral Treg proportions, the impact of MALT1 protease inhibition in an adult organism is still unknown. A previous report in mice using mepazine, an unselective and weak MALT1 protease inhibitor, reported no change in Treg frequency (12).

**FIGURE 1:**
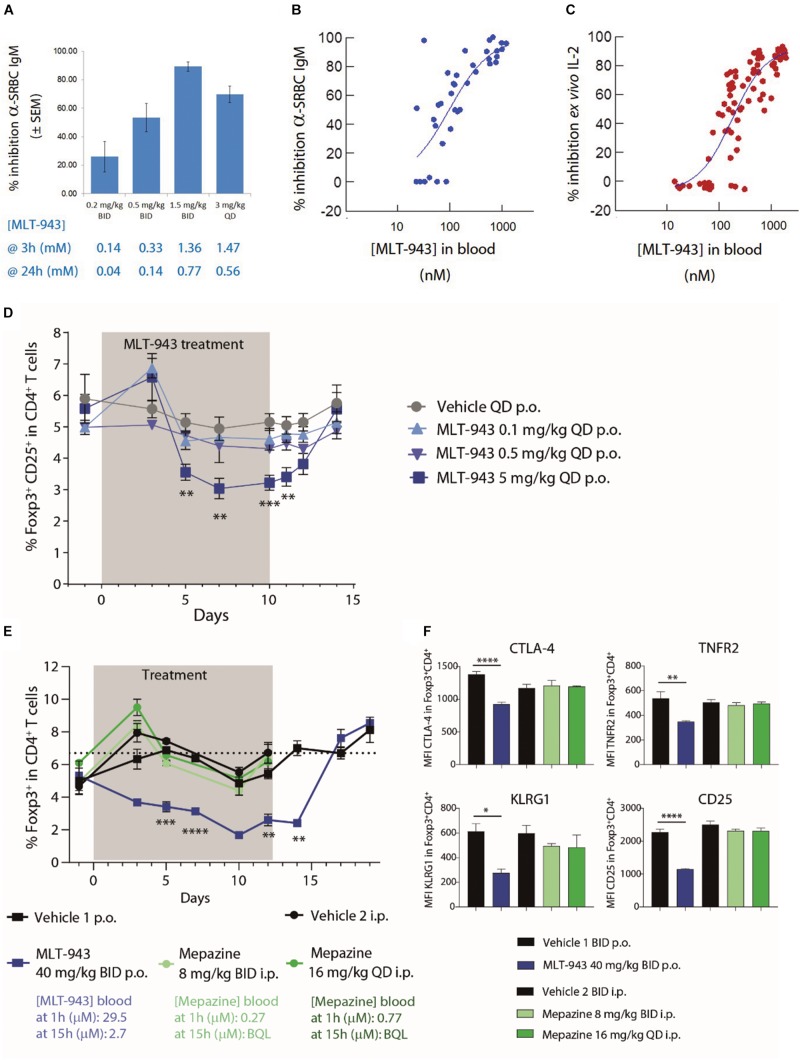
MALT1 protease inhibition by MLT-943 reduces Tregs in rat and mouse. **(A–C)** SRBC immunized rats were treated with MLT-943 at various doses and regimens as indicated in **(A)**. Numbers below the bars indicate 3 and 12 h serum exposure levels of MLT-943 at the given dosing regimen (graph depicts mean ± SEM). **(B)** Graph depicts percent of inhibition of rat anti-SRBC IgM, compared to vehicle group (pooled data of two experiments, *n* = 5 each). The relationship between MLT-943 blood levels at the 12 h trough time point in individual animals with the extent of inhibition of anti-SRBC IgM. **(C)** The relationship between MLT-943 blood levels in individual animals with the extent of inhibition of IL-2 production induced *ex vivo* by stimulation of whole blood with PMA/anti-CD28 antibody. Red lines depict 50% inhibition of anti-SRBC IgM and corresponding MLT-943 concentration. **(D)** Naive rats were treated with MLT-943 for 10 consecutive days. Frequency Foxp3^+^CD25^+^ Tregs in blood during treatment (gray shaded) and after cessation of MLT-943 treatment in blood. **(E,F)** C57BL/6 WT mice were treated with mepazine or MLT-943 for 12 days. Peak (1 h) and trough (15 h) blood compound concentrations are indicated below the legend. BQL: below quantification limit. **(E)** Treg frequency as well as **(F)** functional markers on Tregs in peripheral blood were followed by FACS on day 5 of treatment. Lines depict mean ± SEM. Statistical difference was determined using *t*-tests (one per time point, MLT-943 5 mg/kg QD vs. vehicle: **(D)** and MLT-943 40 mg/kg BID vs. vehicle 1 **(E)**), *p < 0.05, **p < 0.01, ***p < 0.001, ****p < 0.0001.

We first determined the pharmacologically relevant MLT-943 dose range in the rat using the sheep red blood cell (SRBC) model of T cell-dependent immunization. In this model, a minimum residual total concentration (trough level) of ∼0.8 μM (332 ng/ml) of MLT-943 was sufficient to obtain 90% inhibition of antibody production (1.5 mg/kg BID p.o.) (Figures 1A,B). This correlated well with the ∼1 μM (415 ng/ml) blood concentration required *ex vivo* to inhibit PMA/anti-CD28 induced IL-2 production, an assay we validated to predict efficacious dosing *in vivo* (Figure 1C). Consistent with the inhibition of the antibody response to SRBC immunization, prophylactic treatment with MLT-943 (10 mg/kg QD p.o.) in the rat collagen-induced arthritis model suppressed anti-collagen antibody production, fully prevented paw swelling, and normalized joint histology scores (Supplementary Figures S1A–D). In line with the importance of the MALT1 protease for FcgR-induced pro-inflammatory cytokine release (20), therapeutic treatment with MLT-943 (3 or 10 mg/kg QD p.o.) also significantly reduced paw swelling and histological scores in the same model (Supplementary Figures S1E,F). Overall, these data helped identify effective dosing regimens for MLT-943 and confirmed the therapeutic potential of MALT1 protease inhibition.

To assess the effect of MLT-943 on Treg frequency in blood, naïve rats were treated for 10 consecutive days with a pharmacologically effective dose of MLT-943 (5 mg/kg QD p.o.) or suboptimal doses (0.1 and 0.5 mg/kg QD p.o.), and Treg frequency in peripheral blood was assessed over time. As shown in Figure 1D, effective inhibition of MALT1 protease activity using 5 mg/kg of MLT-943 resulted in a progressive reduction in the frequency of Foxp3^+^CD25^+^ Treg cells in circulating CD4^+^ T cells, which was maximal after 7 days of treatment. The reduction in Treg frequency reached ∼50% of the original values, similar to what was reported in MALT1 PD mice (13–16), and persisted throughout the MLT-943 treatment period (Figure 1D). Suboptimal doses of MLT-943 did not impact the Treg frequency suggesting that full and persistent MALT1 protease inhibition is required to alter Treg homeostasis. Of note, discontinuation of MLT-943 treatment after day 10 led to Treg frequency progressively returning to their original values within 4 days (Figure 1D).

Similar findings were observed in mice when treated with an efficacious dose of MLT-943 (40 mg/kg BID p.o.). The frequency of circulating Treg cells rapidly declined upon MLT-943 treatment (Figure 1E). MLT-943 also reduced CTLA-4, TNFR2, KLRG1, and CD25 expression levels on Foxp3^+^ Treg cells (Figure 1F), scoring the importance of MALT1 protease activity for Treg homeostasis and function. Of note, treatment of mice with mepazine using the dosing regimens previously reported by different groups (8 mg/kg BID i.p. or 16 mg/kg QD i.p.) (12, 23, 28) impacted neither the frequency of Treg cells nor the expression of Treg activation markers (Figures 1E,F). These findings highlight the limitations of mepazine for *in vivo* studies aimed at MALT1 protease inhibition, in terms of on-target potency (μM in cellular assays) (17, 19) as well as bioavailability (Figure 1E).

Overall, these data demonstrated that a reduction in circulating Treg frequency is a hallmark of effective MALT1 protease inhibition and that this reduction can be reverted upon treatment cessation.

### Prolonged Treatment With MLT-943 Induces a Severe Immune-Mediated Pathology in the Rat

**FIGURE 2:**
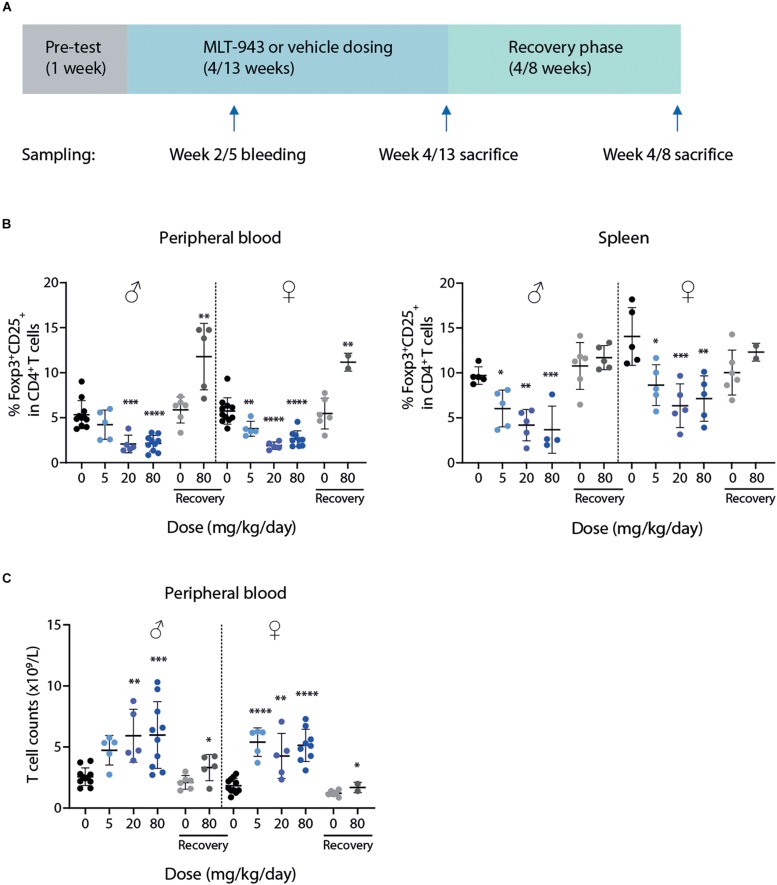
Prolonged pharmacological MALT1 protease inhibition leads to reduced Tregs and increased T cell numbers in rats. **(A)** Outline of 4-week and 13-week GLP toxicity studies including pre-test, treatment and recovery phases. **(B)** Frequency of Foxp3^+^CD25^+^ Tregs in blood (left) and spleen (right) in male and female rats determined by FACS at time of necropsy (day 86 or recovery day 55). **(C)** Total T cell (CD3^+^) counts in blood at time of necropsy (day 86 or recovery day 55). Each dot represents an individual animal. Lines depict mean ± SD. Statistical difference was determined using one-way ANOVA with follow up for significance by multiple comparison tests with Sidak’s correction, *p < 0.05, **p < 0.01, ***p < 0.001, ****p < 0.0001.

MLT-943 was administered by oral gavage to groups of naïve rats at doses of 0, 5, 20 or 80 mg/kg/day for 4 or 13 weeks. Six of the animals at doses of 0 and 80 mg/kg/day were maintained on study for a 4- (in the 4-week study) or 8-week (in the 13-week study) treatment-free recovery period (Figure 2A). Toxicokinetics analysis (Supplementary Tables S2, S3) performed at the end of the treatment period confirmed that full inhibition of MALT1 protease was maintained over the whole treatment period, including at trough levels 24 h post-dose, as residual levels were higher than 415 ng/ml (i.e., ∼1 μM) required for maintaining full efficacy *in vivo* (Figure 1C). A roughly dose proportional exposure increase was observed in rat between 5 and 20 mg/kg after single and multiple oral dosing. Under-dose proportionality was observed in rats at higher oral doses after multiple dosing between 20 and 80 mg/kg/day.

In both the 4- and 13- week rat studies, immunophenotyping of peripheral blood and spleen for lymphocyte subsets showed findings at all doses in both sexes at the end of the dosing phase. In both studies the findings were similar across treatment groups and included principally MLT-943-related changes in the percentages of T cell subsets. Reduced proportions of CD25^+^Foxp3^+^ Tregs (Figure 2B and Supplementary Figures S2A,B) as well as a larger relative proportion and counts of total T cells (Figure 2C and Supplementary Figure S2C) were observed in blood and spleen of rats treated with MLT-943 for either 4 or 13 weeks. Of note, increased T cell counts were associated with an increase in both T helper and Cytotoxic T cell (CTL) populations (data not shown). Importantly, by the end of the respective recovery periods in both studies, the differences in the immunophenotyped subsets in blood and spleen were partially to completely resolved. In the 13-week study, at the end of the 8-week recovery period, percentages of Tregs in blood even increased conversely.

**FIGURE 3:**
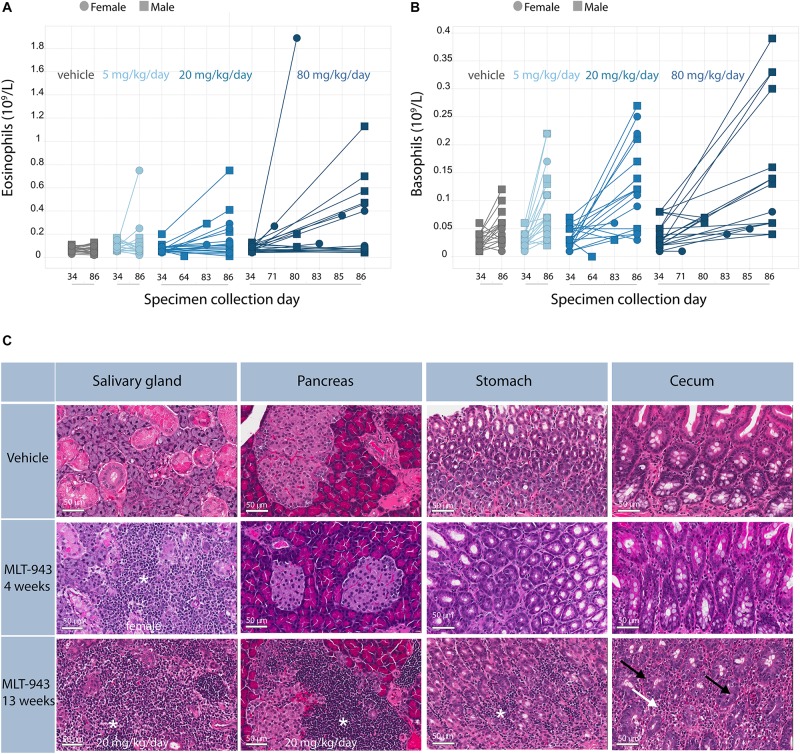
Immune-mediated pathology is induced upon prolonged treatment with MLT-943. **(A)** Eosinophil and **(B)** Basophil counts in peripheral blood, analyzed on day 34 (week 5), day 86 (week 13) or at time point of early euthanasia as indicated. **(C)** Progression of histological alterations in Wistar rats treated with MLT-943 for up to 13 weeks. All animals were males and belonged to the 80 mg/kg/day groups if not stated otherwise in the picture. Sections were taken at necropsy day 27 (week 4) or day 86 (week 13; except for cecum 13 week MLT-943, early death day 80) and stained with H&E. Mononuclear cell infiltration in salivary glands, pancreas and stomach (*). Mixed cell infiltration (white arrow), globule leukocytes (black arrow) in stomach/cecum (higher resolution pictures in Supplementary Figure S3A).

Overall, oral administration of MLT-943 to rats for 4 weeks at doses up to 80 mg/kg/day was well tolerated, however, animals treated with all dose levels of MLT-943 (≥5 mg/kg/day) displayed low-grade lymphocytic infiltration in kidneys and lacrimal glands associated with an increase in lymphocyte and monocyte counts, which partially reversed during the recovery period. In contrast, during the course of the 13-week study, severe clinical symptoms occurred in several rats dosed at 20 and 80 mg MLT-943/kg/day, starting on day 64 (week 9) of treatment. Impaired general condition, abdominal distension, body weight loss and/or decreased body weight gain and decreased food consumption generally preceded animal euthanasia. The main cause of moribundity was degenerative and inflammatory gastro-intestinal (GI) lesions, with ulcerative and inflammatory skin lesions in individual animals. Additionally, two animals displayed lesions of the endocrine pancreas (atrophy, mononuclear cell infiltration), which correlated in clinical biochemistry with a marked increase in serum glucose concentration and a marked decrease in serum bicarbonate concentration, and was consistent with secondary ketoacidosis (data not shown). In surviving animals, marked changes in white blood cells were observed on day 86 (week 12) starting at the lowest dose of 5 mg/kg/day. These were characterized by increased counts of neutrophils, lymphocytes, monocytes, eosinophils, and basophils, as exemplified here by eosinophils (Figure 3A) and basophils (Figure 3B). White blood cell increases at the end of the treatment period were associated with the mononuclear and mixed inflammatory cell infiltrates described histologically in numerous organs including the gastrointestinal tract and pancreas (Figure 3C and Supplementary Figure S3). Histopathologically, alterations in the gastro-intestinal tract were present at >5 mg/kg/day, showed some dose-dependency and included mucosal degeneration/regeneration, marked increase of globule leukocytes and mixed inflammatory cell infiltrates with many eosinophils and immature cells, probably mast cells (Figure 3C and Supplementary Figure S3A). Mononuclear cell infiltrates were present in several organs/tissues at >5 mg/kg/day with similar severity across treatment groups (Figure 3C and Supplementary Figure S3B). In addition, degenerative and subsequent regenerative changes were present in numerous animals, especially in liver, the exocrine and endocrine pancreas, stomach, and skin. Lymph node activation, bone marrow hypercellularity (particularly affecting the eosinophilic lineage) and increased extramedullary hematopoiesis in the spleen might have resulted from inflammatory changes in other organs/tissues. However, a direct effect of MLT-943 cannot be excluded. After the recovery period in surviving animals, hematological and microscopic changes were reversible or, if not, showed a tendency toward reversibility (Table 2).

**TABLE 2:** Partial recovery of histological findings in selected tissues after 13 weeks treatment with MLT-943 followed by an 8-week recovery period – incidence and mean severity of affected animals.

Treatment/finding	0 mg/kg after 13 weeks		0 mg/kg after recovery		MLT-943 80 mg/kg after 13 weeks		MLT-943 80 mg/kg after recovery	
				
Sex	Male	Female	Male	Female	MALE	Female	Male	Female
No. animals/group	10	10	6	6	11*	14*	5	2
Degeneration/regeneration, stomach	0 (−)	0 (−)	0 (−)	0 (−)	4 (1.3)	2 (1.5)	0 (−)	0 (−)
Mononuclear cell infiltration, stomach	0 (−)	0 (−)	0 (−)	1 (2.0)	8 (1.8)	8 (1.4)	3 (1.3)	0 (−)
Globule leukocytes, stomach	4 (1.0)	3 (1.0)	2 (1.0)	3 (1.0)	11 (2.1)	12 (2.0)	3 (1.0)	1 (3.0)
Degeneration/regeneration, cecum	0 (−)	0 (−)	0 (−)	0 (−)	6 (2.7)	6 (2.0)	0 (−)	0 (−)
Mixed cell infiltration, cecum	0 (−)	0 (−)	0 (−)	0 (−)	10 (2.1)	9 (2.1)	1 (1.0)	1 (1.0)
Globule leukocytes, cecum	0 (−)	0 (−)	0 (−)	0 (−)	10 (2.5)	9 (2.7)	0 (−)	0 (−)
Mononuclear cell infiltration, parotid salivary gland	0 (−)	0 (−)	1 (1.0)	1 (1.0)	5 (1.6)	4 (1.0)	3 (1.0)	1 (1.0)
Mononuclear cell infiltration, pancreas	2 (1.0)	0 (−)	2 (1.0)	0 (−)	5 (1.4)	7 (1.6)	4 (1.0)	2 (1.0)

Severity grading: 1, minimal; 2, mild; 3, moderate; 4, marked; (), mean severity of affected animals/group * including early sacrifices/deaths.

In conclusion, treatment with MLT-943 caused a reduction in Treg and an increase in total T cell counts, in both 4- and 13-week rat toxicity studies at all dose levels. While a 4-week treatment was well tolerated, with low-grade organ lymphocytic infiltration observed at all dose levels, longer treatment induced severe immune-mediated pathology in multiple organs, with clinical onset starting around week 9, and hematology changes detected as early as week 5 of treatment.

### MLT-943-Induced Inflammation Correlates With Activation of GI Tract-Resident Cells

**FIGURE 4:**
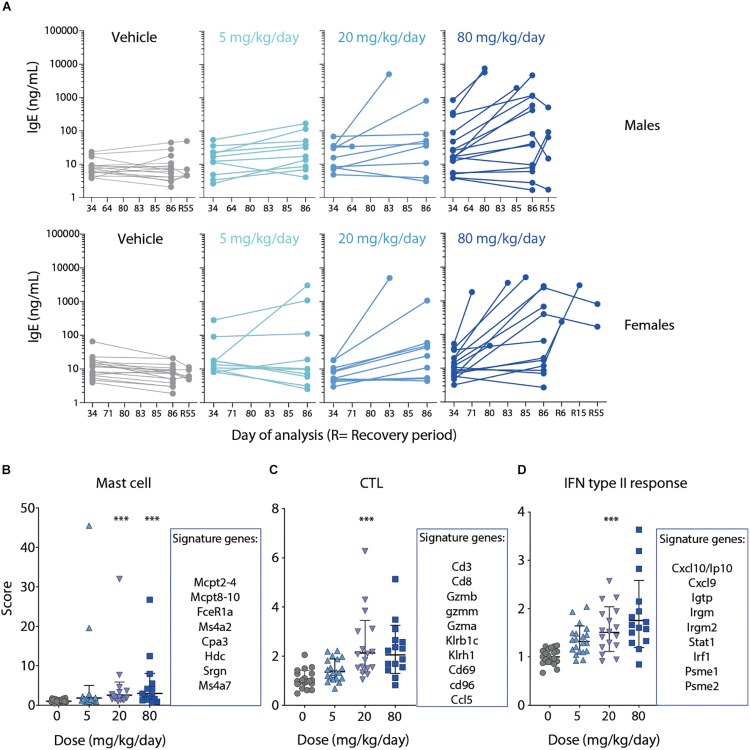
Severe GI inflammation in rats correlates with elevated IgE, mast cell and CTL signatures. **(A)** Serum levels of IgE analyzed on day 34 (week 5), day 86 (week 13), day 55 (week 8) of recovery phase or at time point of early euthanasia as indicate in male (upper panel) and female rats (lower panel). **(B–D)** Gene expression analysis of jejunum tissue harvested at necropsy of the 13-week GLP study. Top genes defining the respective **(B)** Mast cell, **(C)** CTL or **(D)** IFN type II response gene signatures are listed next to the graphs. Lines depict geometric mean ± geometric SD. Statistical difference was determined using one way ANOVA (Kruskal-Wallis test), ***p < 0.001.

Serum IgE concentration was analyzed during the 13-week study dosing period (day 34 and day 86), during the recovery phase (day R55), and at the time of unscheduled euthanasia. MLT-943-related increases in serum IgE concentration were observed at doses ≥5 mg/kg/day in both male and female rats on day 86, and in male rats also on day 34 (Figure 4A). Values in prematurely euthanized animals were markedly elevated. Partial resolution was seen in both genders at the end of the recovery phase (Figure 4A).

Gene expression profiling of jejunum samples was performed to characterize the molecular changes induced by MLT-943 when administered to rats for 13 weeks. MLT-943 induced a dose-dependent up-regulation of a mast cell gene signature (Figure 4B). The up-regulation was statistically significant compared to vehicle controls at doses ≥20 mg/kg/day. There was high inter-animal variability, with some animals showing very high expression of the signature already at 5 mg/kg/day (Figure 4B). Additionally, MLT-943 up-regulated CTL (Figure 4C) and interferon (IFN) (Figure 4D) gene signatures. The IFN gene signature contained transcripts mainly induced by gamma interferon, thus reflecting a type II interferon response. The CTL and IFN signatures were both most prominently upregulated in the 20 and 80 mg/kg/day groups. The lack of clear dose-effect relationship for the CTL gene signature may result from a limited effect, the inherent inter-animal variability, or sampling differences. Of note, T cells and IFNγ were described as major drivers of the pathology occurring in MALT1 PD animals (14, 25) and we observed increased T cell numbers in blood after 4- and 13-week MLT-943 treatment (Figure 2C and Supplementary Figure S2C).

Collectively, the systemic inflammatory pathology induced by long-term MLT-943 treatment in rats resulted in activation and/or recruitment of both innate and adaptive immune cells in the GI tract. These data are in line with the elevated serum IgE and the systemic T cell activation observed previously in the MALT1 PD mouse model as a result of the reduced regulatory T cell compartment (13–16, 25).

### Absence of Thymic Activity Does Not Prevent MLT-943-Driven Treg Reduction and Immunopathology

The progressive multi-organ pathology described above was reminiscent of the inflammatory disease observed in MALT1 PD mice (13–16). This suggested that dysregulation of immune homeostasis subsequent to a reduction in Treg frequency could be the cause of the immune alterations upon MALT1 protease inhibition.

**FIGURE 5:**
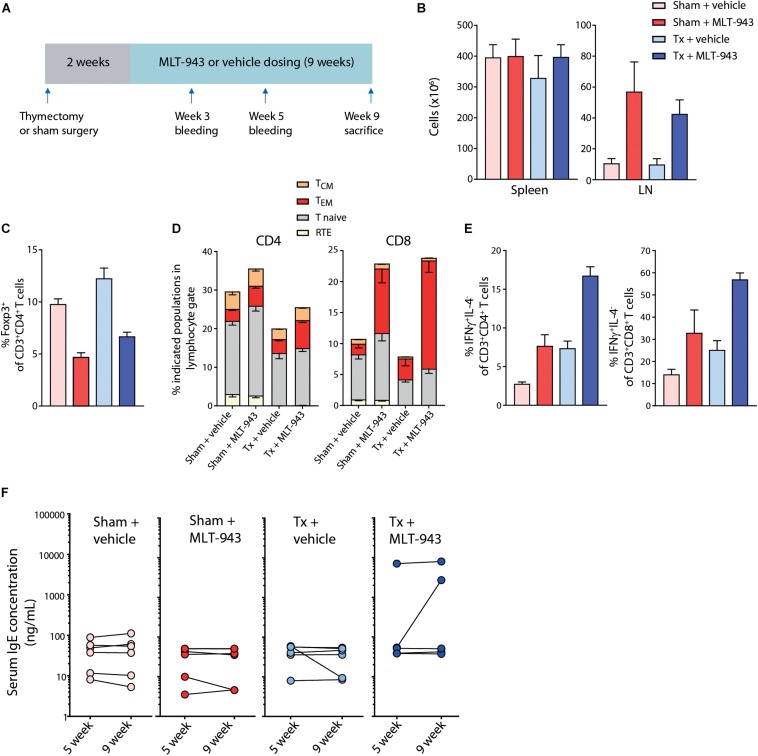
Thymectomy does not prevent the MLT-943-associated Treg reduction and immunopathology. **(A)** Outline of the 9-week thymectomy study including surgery, regeneration time after surgery (2 weeks) and 9 weeks MLT-943 or vehicle dosing phase. **(B–E)** Tissues and peripheral blood from male and female rats were harvested at necropsy (day 63) and analyzed by standard hematology (cell counts) or FACS. **(B)** Cell counts in spleen and LN in the indicated groups. **(C)** Frequency of Foxp3^+^ Tregs in blood in the indicated groups. **(D)** Frequency of indicated T cell subpopulations in blood. **(E)** Percentage of IFNγ-producing, IL-4-negative CD4 and CD8 T cells isolated from blood, detected by intracellular FACS staining after 4 h PMA/Ionomycin stimulation *ex vivo*. Graphs depict means ± SEM. **(F)** Serum IgE levels in male and female rats at day 35 (week 5) and day 63 (week 9) of MLT-943 or vehicle treatment.

Thymic Treg development is drastically reduced in MALT1 PD mice (13–15) and limited thymic output of Treg cells may contribute to the peripheral Treg reduction observed upon congenital MALT1 protease inactivation or pharmacological inhibition. In humans, the thymic mass and activity markedly diminishes during the first two decades of life (39). While the adult human naive T cell pool is maintained mainly through peripheral T cell division, naive T cells in mice are almost exclusively sustained by thymic output throughout their lifetime (40). Given the rapid Treg reduction observed in mice and rats upon MALT1 protease inhibition, we addressed the relevance of the effects on thymic function in the progressive pathology observed upon long-term MLT-943 administration. To this end, naïve adult rats were thymectomized (Tx) before dosing with 20 mg/kg/day p.o. MLT-943 for 9 weeks (Figure 5A). To confirm the efficiency of the thymectomy and to identify different subset of T cells, we implemented a multi-color FACS panel allowing for identification of Recent Thymic Emigrants (RTE), Naive, Effector Memory (T_*EM*_) and Central Memory (T_*CM*_) CD4 and CD8 T cell subsets in rats (Supplementary Figures S4A,B) based on markers reported previously (41). In pilot experiments, we confirmed that 1 week after thymectomy, the RTE CD4 and CD8 T cell populations were already efficiently depleted from the peripheral T cell pool (Supplementary Figure S4C). At 9 weeks of treatment, blood, spleen and mandibular lymph nodes (LNs) were collected and cells analyzed by flow cytometry. While the splenic cellularity was impacted neither by the thymectomy procedure nor by MLT-943 treatment, mandibular LNs displayed a significant increase in cellularity following MLT-943 treatment, which was not affected by the thymectomy procedure (Figure 5B). Analysis of the T cell compartments showed a ∼50% reduction in Treg frequency in the CD4^+^ T cell compartment of MLT-943-treated euthymic animals (Figure 5C). Of note, thymectomized rats displayed a similar Treg reduction upon MLT-943 treatment indicating that MALT1 protease inhibition impacted Treg homeostasis independently of its effects on thymic Treg development or output (Figure 5C and Supplementary Figures S5A,D). Consequent to the reduced Treg frequency, the T_*EM*_ compartment was increased within CD8^+^ T cells (Figure 5D and Supplementary Figures S5B,E) and both CD4^+^ and CD8^+^ T cells in MLT-943-treated groups produced more IFNγ upon *ex vivo* stimulation (Figure 5E and Supplementary Figures S5C,F). All these MLT-943-related effects occurred independently of the presence of an intact thymus.

Similarly, thymectomy did neither prevent the development nor influence the severity of histopathological and hematological alterations. The MLT-943-related increase in serum IgE concentration was observed in 3 out of 12 animals, of which all were thymectomized (Figure 5F). Increased incidence or severity of mononuclear cell infiltration was seen in glandular tissues and kidneys in all groups treated with MLT-943 (both intact and thymectomized rats) compared to vehicle controls, and individual MLT-943 treated rats showed intestinal changes similar to those observed in the 13-week toxicity study (Table 3). Of note, the animals most severely affected by gastrointestinal alterations were those with the highest serum IgE concentrations and increased leukocyte counts.

**TABLE 3:** Histological alterations in thymectomized or sham-operated Wistar rats treated with MLT-943 or vehicle for up to 9 weeks.

Treatment finding	Sham-operated, vehicle	Sham-operated, MLT-943	Thymectomized, vehicle	Thymectomized, MLT-943
No, animals/group	12	11	11	12
Mononuclear cell infiltration, salivary gland	0	8	0	1
Mononuclear cell infiltration, lacrimal gland	*2*	7	1	9
Mononuclear cell infiltration, kidney	0	7	1	8
Mononuclear cell infiltration, pancreas	1	7	0	8
Mononuclear cell infiltration, stomach	0	7	0	4
Degeneration/regeneration duodenum/cecum	0	1	0	3
Mixed cell infiltration, small and/or large intestine	0	6	0	4
Globule leukocytes, small and/or large intestine	0	5	0	3

*Numbers indicate the amount of animals per group showing alterations.*

In conclusion, pharmacological MALT1 inhibition disrupted Treg homeostasis and function independently of effects on thymic Treg output.

### Prolonged Treatment With MLT-943 in the Dog Also Leads to a Reduction of Tregs and to Immunological Disorders

**FIGURE 6:**
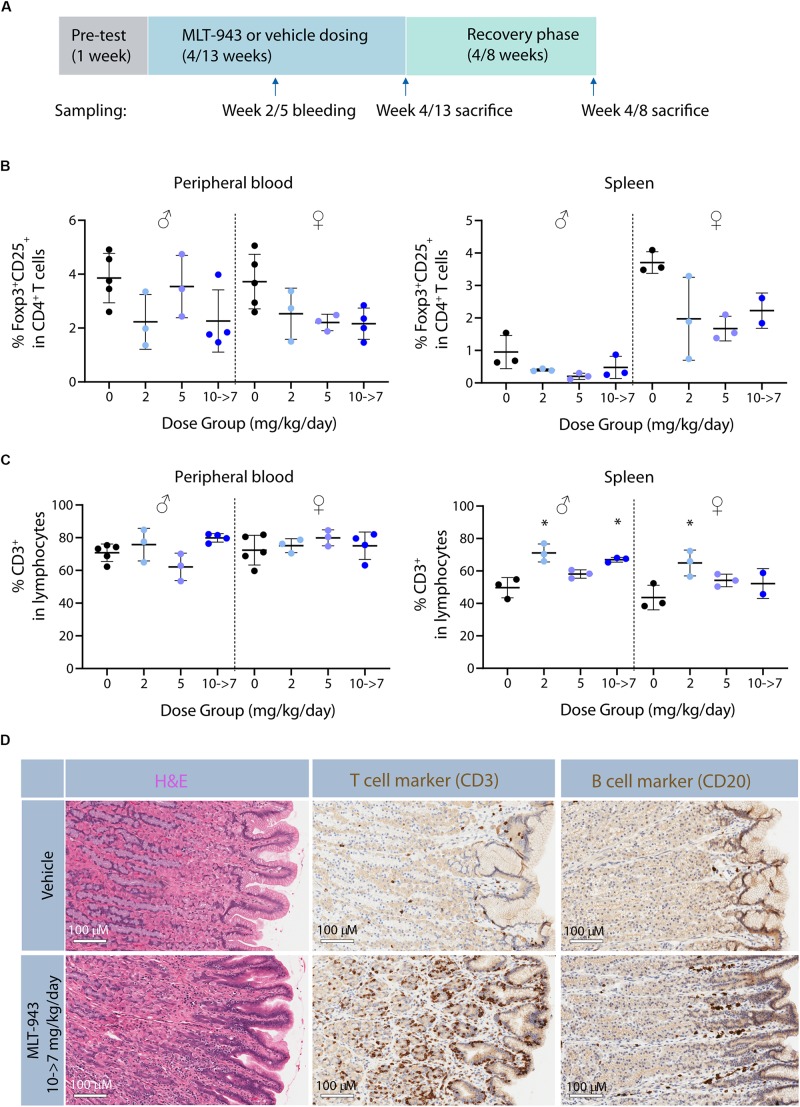
Prolonged pharmacological MALT1 protease inhibition leads to reduced Tregs in dogs. **(A)** Outline of 4-week and 13-week GLP toxicity studies including pre-test, treatment and recovery phases. **(B)** Frequency of Foxp3^+^CD25^+^ Tregs and **(C)** CD3^+^ T cells in blood and spleen in male and female beagle dogs determined by FACS at time of necropsy (day 86 or recovery day 55). Each dot represents an individual animal. Lines depict mean ± SD. Statistical difference was determined using one-way ANOVA with follow up for significance by multiple comparison tests with Sidak’s correction,*p < 0.05. **(D)** Histological alterations in stomach of beagle dogs treated with MLT-943 for up to 13 weeks. All animals were females and belonged to either the vehicle or the 10→7 mg/kg/day group. Sections taken at necropsy day 86 and stained with H&E (left panel), anti-CD3 (middle panel), or anti-CD20 (right panel).

MLT-943 was administered by oral gavage to groups of Beagle dogs (3 or 5/sex/group) at doses of 0, 0.5, 2, 5 mg/kg/day p.o. for at least 4 weeks; or 2, 5, and 10→7 mg/kg/day for at least 13 weeks. Two of the animals in the control and high-dose groups were maintained on study for a recovery period (4 weeks in the 4-week study or 8 weeks in the 13-week study, Figure 6A).

In the 4-week dog study, decreases in peripheral blood and spleen Treg cells (CD25^+^FoxP3^+^, Supplementary Figure S6) were observed at the mid- and high-doses (2 and 5 mg/kg/day) in both sexes at the end of the dosing phase (Supplementary Figures S7A,B). In blood this was observed as early as after 9 days of treatment start. Additionally, MLT-943 treatment resulted in lower total B cell proportions at both doses in blood and spleen, compared to controls (data not shown). These changes in spleen were matched by decreases in Treg and B cell counts/gram. However, no difference in the proportions of total T cells, T helper or CTLs in blood or spleen after 4 weeks of dosing could be clearly detected among the dogs in any dose group (Supplementary Figure S7C). Results in animals evaluated at the end of the recovery phase indicated partial to complete resolution of these changes. In the 13-week dog study, MLT-943-related findings occurred at all doses, impacting both Treg and total T cell proportions (Figures 6B,C). Findings included lower proportion of blood Treg cells, in males at ≥2 mg/kg/day at week 5 and 13 and in females at ≥5 mg/kg/day at week 13 (Figure 6B). Again, lower B cell proportions were observed in both genders at all doses at weeks 5 and 13 (data not shown). No clear differences in total T cell, T helper or CTL subsets were noted in blood (Figure 6C and data not shown). Nevertheless, these results together with the higher T:B cell count ratio in blood of these dogs suggested the higher total T cell proportions could have accounted for the proportionally lower B cells at all MLT-943 doses for both genders relative to controls. Accordingly, splenic lymphoid subsets showed greater total T cell, T helper and CTL cell subsets and lower Tregs and B cell proportions in both sexes at all MLT-943 doses at the end of the dosing phase relative to vehicle controls (Figure 6C and data not shown). Lower splenic Treg counts and higher T:B cell ratios for total counts (as in peripheral blood) were also observed.

MLT-943 was well tolerated in the 4-week toxicity study, with no treatment-related clinical signs, changes in body weight and food consumption, or any anatomic pathology findings. A roughly dose proportional exposure increase was observed between 0.5 and 10 mg/kg after single and multiple oral dosing. Based on exposure levels (Supplementary Table S4) compared to whole blood activity (Table 1), together with the lack of effect on regulatory T cell counts (data not shown), the lowest dose of 0.5 mg/kg/day did not achieve full MALT1 inhibition over the treatment period. Consequently, doses were increased in a subsequent 13-week toxicity study (Supplementary Table S5). During that study, MLT-943 related clinical signs were observed at all doses, starting prominently from week 4 onward and including several fecal changes (soft or mucoid feces, diarrhea or feces with bloody content). Loss of body weight/reduced body weight gain and reduced food consumption were observed in the highest dose group. Treatment-related moribundity leading to early euthanasia occurred in two high dose animals: one female on day 50, and one male on day 69. Due to the severity of the clinical signs, the highest dose of 10 mg/kg/day was reduced to 7 mg/kg/day from day 55 onward. The main microscopic finding in the 13-week study observed across all MLT-943 treatment groups was an infiltration of mononuclear cells in the stomach, the large intestine and the liver, which had not been observed in the 4-week toxicity study. To characterize the nature of the mononuclear infiltrates, immunohistochemical analyses were performed on selected high-dose animals. In the stomach, the majority of the mucosal mononuclear infiltrate consisted of CD3^+^ T cells rather than of CD20^+^ B cells (Figure 6D). In the liver, the perivascular infiltrate was composed of similar numbers of CD3^+^ T cells and CD20^+^ B cells while in the sinusoids, CD3^+^ T cells seemed to be present in higher numbers when compared to stainings of control animals (data not shown). Changes in clinical pathology considered consistent with the presence of inflammation and immunomodulation included increased peripheral blood neutrophil and monocyte counts, increased plasma fibrinogen concentrations, and decreased serum albumin concentrations.

In summary, MLT-943 treatment of dogs led to Treg reduction of similar magnitude as seen in the treated rats (above) and in MALT1 PD mice (13–16). While a 4-week MLT-943 treatment was well tolerated without significant findings, prolonged treatment led to a severe, immune-mediated pathology with clinical features of colitis and infiltration of mononuclear cells in the stomach, the large intestine, and the liver.

## Discussion

Given its central role downstream of multiple immune receptors, the MALT1 protease is an attractive therapeutic target for a variety of indications spanning from autoimmunity to lymphomas. While this view is supported by several preclinical studies using MALT1 PD animals or tool MALT1 inhibitors, the spontaneous multi-organ autoimmune disease that develops in MALT1 PD animals has raised concerns about the safety of pharmacological inhibition of the MALT1 protease. Here, we have addressed this safety question using MLT-943, a novel potent and highly selective MALT1 protease inhibitor. Our study has shown the central role of MALT1 for immune homeostasis maintenance in adult individuals highlighting the risk associated with potential testing of pharmacological MALT1 inhibitors in humans.

The high selectivity and potency of MLT-943 was supported by a wide range of enzymatic assays (Supplementary Table S1, Table 1, and Figure 1). We showed that MLT-943 suppressed antibody production upon SRBC immunization in rats, with a potency matching *ex vivo* inhibition of IL-2 production by stimulated T cells. In contrast to MLT-943, the potency and/or selectivity of MALT1 compounds used so far in *in vivo* models, e.g., MI-2 or mepazine, has remained questionable (17, 42). We provided compelling comparative evidence that treatment of naive mice with reported doses of mepazine did not impact the frequency of Tregs, contrasting with pharmacologically effective doses of MLT-943 that promoted a rapid and progressive decrease in the frequency of circulating Foxp3^+^ Treg cells and in Treg CTLA-4, TNFR2, CD25, and KLRG1 levels. In fact, the reduction in Treg frequency observed with MLT-943 emerged as a clear pharmacodynamic marker of MALT1 activity. This finding has important implications and suggests that previous *in vivo* data obtained with tool MALT1 inhibitors such as mepazine have to be interpreted cautiously. The lack of effect of mepazine on Tregs *in vivo* is likely a consequence of a weak potency and of limited blood levels for achieving efficient and sustained MALT1 protease inhibition. Notably, the effect on Treg cells observed upon MLT-943 treatment was rapidly reversed upon treatment cessation, demonstrating the pivotal role of the MALT1 protease in the homeostasis of the Treg compartment.

MLT-943 was administered orally to rat and dog species in 4- and 13-week toxicity studies. Inhibition of MALT1 with MLT-943 in adult animals led to progressive development of an autoimmune phenotype similar to the IPEX-like disease observed in mice with congenital deficiency in MALT1 protease activity. Furthermore, MLT-943 treatment reduced Treg numbers and led to immune alterations and signs of the IPEX-like disease even in thymectomized rats, demonstrating that peripheral Treg homeostasis and function, and not just thymic Treg development, is critically dependent on MALT1 protease activity.

MALT1 protease inhibition/deficiency appears to be beneficial in multiple preclinical disease models (13–15, 18, 19, 23, 24). Here we showed that prophylactic treatment with MLT-943 prevented anti-collagen antibody generation and joint inflammation in the rat collagen-induced arthritis model. MLT-943 also inhibited joint swelling when tested therapeutically, confirming the pivotal role of the MALT1 protease in FcgR-mediated inflammation (20). Despite the efficacy of MALT1 protease inhibition in these short-term models, the progressive immune alterations and multiorgan disease observed upon long-term treatment with MLT-943 highlighted the risks and challenges associated with potential clinical application of MALT1 protease inhibitors. Based on our data, treatment of chronic autoimmune conditions, which relies on long-term, sometimes life-long therapies, would raise important safety concerns. Whether MALT1 inhibitors might be tolerated if used more acutely in certain indications remains to be considered cautiously. Because the effect on Tregs is rapid and despite its reversibility, the window between benefit (on the disease parameters) and risk (Treg-dependent outcomes) may be narrow and difficult to control. In addition, the actual window in humans remains unknown and clinical work would require the identification of appropriate biomarkers for accurate monitoring of immune alterations. Additional factors such as the underlying clinical pathology and health status of treated patients as well as potential co-medication treatments, may also challenge potential clinical evaluations of MALT1 protease inhibitors. The suggested combination of MALT1 inhibitors with checkpoint inhibitors like anti-PD-1 antibodies (28, 29) might accelerate the appearance of side effects by impacting additional immunoregulatory networks. T cells and IFN-γ are major drivers of the progressive pathology observed in MALT1 PD mice (14, 25). Combination with T cell depleting agents (25) or other immunomodulatory drugs dampening the dominant pathogenic cell subsets and pathways (e.g., IFNγ receptor signaling) might delay or prevent the development of the severe immune alterations observed upon long-term MALT1 protease inhibition but might also introduce the risk of excessive immunosuppression. Potential treatment of lymphomas with a MALT1 inhibitor has been contemplated based on numerous studies showing that MALT1 protease function is a key driver for proliferation of e.g., ABC-DLBCL lymphomas (10, 11) and MALT1 protease inhibitors are now entering clinical trials for these indications (43). Overall, our work highlighted the risks and challenges of pursuing with a MALT1 inhibitor into the clinic, and strongly advocates for careful monitoring of patients, who may enter trials with an already impaired health status.

Alternative treatment strategies including intermittent dosing or acute application of MLT-943 would deserve further evaluation given the pivotal role of MALT1 in multiple immunomodulatory pathways. In addition, given the reduced IPEX-like disease observed with MALT1 KO animals (13–16) a paradigm to abrogate both MALT1 protease and scaffolding function might still remain attractive to circumvent the toxicities observed upon selective inhibition of the MALT1 protease.

Despite an excellent selectivity profile, we cannot fully exclude that some of the findings observed in this study might be related to MLT-943 itself in addition to MALT1 protease inhibition. Studies using different classes of compounds would be warranted to investigate this possibility. However, and very clearly, most of the effects and immune alterations observed with MLT-943 were consistent with the biological findings uncovered by the study of MALT1 PD mice or reported in MALT1 deficient patients.

## Data Availability Statement

Gene expression data have been deposited in the ArrayExpress database at EMBL-EBI (www.ebi.ac.uk/arrayexpress) under accession number E-MTAB-8990.

## Ethics Statement

The animal study was reviewed and approved by the Swiss Federal and Cantonal Authorities, US and EU animal health regulations.

## Author Contributions

TC, NR, and FB conceptualized and designed the work and wrote the manuscript. KM collected all data, prepared the figures, and wrote the manuscript. UJ performed/supervised the histopathology evaluation and contributed to the manuscript. ET performed the gene expression analyses and contributed to the manuscript. ES performed the histopathology evaluation and contributed to the manuscript. TR-S performed the Treg measurements in the dog and contributed to the manuscript. HM coordinated safety endpoint analysis in pharmacology and toxicology studies. SN performed the rat and dog whole blood assays. JL performed the thymectomies. AS and JQ supervised the MLT-943 related activities. DW supervised the toxicity studies and immunophenotyping analysis and contributed to the manuscript. MB supervised the SRBC experiments. AL-E supervised the rat collagen-induced arthritis experiments. DL supervised the hematology part and contributed to the manuscript. KB supervised the pharmacokinetics and toxicokinetics work, and contributed to the manuscript. CB, BR, and ML contributed to PK and modeling studies.

## Conflict of Interest

All authors are or were employees and/or shareholders of Novartis Pharma AG.
