# Supplementary material for: Pharmacological Inhibition of MALT1 Protease Leads to a Progressive IPEX-Like Pathology
Source: Front Immunol. 2020 Apr 30;11:745. doi: 10.3389/fimmu.2020.00745 (PMC7203682; doi:10.3389/fimmu.2020.00745)
Supplement: Supplementary file 1 [file Data_Sheet_1.PDF]

Supplementary Table 1: Biochemical assays to determine the inhibitory potency of MLT-943 for a panel of enzymes.

Compound concentration:
The compound was tested in each assay as a concentration-response curve (12 concentrations, half-log dilutions starting at 30 μM, or 300 μM for the BSEP assay)

Modality	HTRF: Homogeneous Time Resolved Fluorescence	METHODS						
B binding<br>F functional (cell-based)<br>E enzymatic	**Target name**	**membrane<br>tissue<br>host cell**	**Ligand/ substrate**	**Max effect (active control / inhibition or activation)**	**Incubation (time/ temp)**	**Protocol**	**Target ID**	**DRC data EC_{50}/IC_{50} (μM)**
B	Adenosine 1 receptor binding assay, human	CHO-K1	[3H]-NECA	DPCPX 10 μM	15 h / RT	SPA	hr Ad1 binding	> 30
F	Adenosine 1 receptor antagonist assay	CHO-K1	CPA (0.0092 μM)	DPCPX 10 μM	15 min / 30°C	cAMP	hr Ad1 anta	11
B	Adenosine 2A receptor binding assay, human	HEK-293	[3H]-CGS21680	CGS21680 10 μM	10 h / RT	SPA	hr Ad2A binding	25
F	Adenosine 2A receptor antagonist assay	HEK-293	NECA (0.0012 μM)	DPCPX 10 μM	10 min / 37°C	cAMP	hr Ad2A anta	13.4
F	Prostanoid FP, IP1 receptor antagonist assay	HEK-293 EBNA1 cells	Cloprostenol 0.0014 μM	AL88100 100 μM	30 min / 37°C	Filtration	hr FP anta	7.6
B	Adenosine 3 receptor binding assay, human	CHO-K1	[125I]-AB-MECA	IB-MECA 10 μM	15 h / RT	SPA	hr Ad3 binding	29
F	Alpha2A receptor antagonist assay	CHO-K1	(-)norepinephrine 0.3 μM	RX821002 10 μM	15 min / RT	Ca flux/ATP	hr Al2A antag	14
F	Alpha1A receptor agonist assay	CHO-K1	n/a	A-61603 1 μM	2 min / RT	Ca flux	hr Al1A ag	> 30
F	Alpha1A receptor antagonist assay	CHO-K1	A-61603 0.1 μM	WB-4101 1 μM	15 min / RT	Ca flux	hr Al1A antag	> 30
F	Alpha2A receptor agonist assay	CHO-K1	n/a	(-)norepinephrine 1 μM	2 min / RT	Ca flux/ATP	hr Al2A ag	> 30
B	Adrenergic Alpha 2B receptor assay : human	CHO-K1	[3H]-RX821002	RX821002 10 μM	20 h / RT	SPA	hr Alpha2B	> 30
B	Adrenergic Alpha 2C receptor assay : human	Sf9	[3H]-RX821002	RX821002 10 μM	20 h / RT	SPA	hr Alpha2C	> 30
B	Beta1 adrenergic receptor assay : human	Sf9	[3H]-dihydroalprenolol	Alprenolol 10 μM	10 h / RT	SPA	hr Beta1	> 30
F	Beta2 adrenergic receptor agonist assay	CHO-K1	n/a	Isoproterenol 3 μM	30 min / RT	cAMP	hr Beta2 ag	> 30
B	Angiotensin II AT1 receptor binding assay,	CHO-K1	[125I]-Sar1,Ile8-Ang II	Sar1,Ile8-Ang II 1 μM	10 h / RT	SPA	hr AT1	> 30
F	Cannabinoid 1 receptor agonist assay	CHEM 1	n/a	CP55940 1 μM	2 min / RT	Ca flux	hr CB1 ag	> 30
F	Cannabinoid 1 receptor antagonist assay	CHEM 1	CP55940 0.25 μM	AM251 300 nM	15 min / RT	Ca flux	hr CB1 antag	> 30
B	Cholecystokinin A receptor binding assay,	1321N1	[3H]-CCK-8-sulfated	CCK-8-sulfated 10 μM	5 h / RT	SPA	hr CCKa	> 30
F	Dopamine D1 receptor agonist assay	CHO-K1	n/a	SKF38393 0.3 μM	30 min / RT	cAMP	hr D1 ag	> 30
F	Dopamine D1 receptor antagonist assay	CHO-K1	SKF38393 6 nM	LE300 1 μM	30 min / RT	cAMP	hr D1 antag	> 10
B	Dopamine D2 receptor assay: human	Sf9	[3H]-Spiperone	Haloperidol 10 μM	20 h / RT	SPA	hr D2	> 30
B	Dopamine D3 receptor assay: human	CHO-K1	[3H]-Spiperone	(+)butaclamol 10 μM	20 h / RT	SPA	hr D3	> 30
B	Endothelin A receptor binding assay, human	CHO-K1	[125I]-Endothelin-1	Endothelin-1 1 μM	20 h / RT	SPA	hr ETa	> 30
B	Ghrelin receptor binding assay, human	CHO-K1	[125I]-Ghrelin	hGhrelin 1 μM	2 h / RT	SPA	hr GHS	> 30
B	Hitamine type 1 receptor assay: human	CHO-K1	[3H]-Pyrilamine	Pyrilamine 1 μM	20 h / RT	SPA	hr H1	> 30
B	Hitamine type 3 receptor assay: human	CHO-K1	[3H]-met histamine	Clobenpropit 1 μM	15 h / RT	SPA	hr H3	> 30
F	Serotonin 5HT1A receptor agonist assay	HeLa	n/a	8-OH-DPAT 1 μM	3 min / RT	Ca flux/ATP	hr 5HT1A ag	> 30
F	Serotonin 5HT1A receptor antagonist assay	HeLa	8-OH-DPAT 0.01 μM	(S)-WAY 100135 1 μM	15 min / RT	Ca flux/ATP	hr 5HT1A antag	> 30
F	Serotonin 5HT2A receptor agonist assay	CHO-K1	n/a	5-HT 1 μM	2 min / RT	Ca flux	hr 5HT2A ag	> 30
F	Serotonin 5HT2A receptor antagonist assay	CHO-K1	5-HT 0.004 μM	Ketanserin 1 μM	15 min / RT	Ca flux	hr 5HT2A antag	23
F	Serotonin 5HT2B receptor agonist assay	CHO-K1	n/a	BW723C86 5 μM	2 min / 37°C	Ca flux	hr 5HT2B ag (n=3)	> 30
F	Serotonin 5HT2B receptor antagonist assay	CHO-K1	BW723C86 0.035 μM	SB221284 10 μM	30 min / 37°C	Ca flux	hr 5HT2B antag (n=3)	1.4
B	Serotonin 5HT2C receptor assay, human	1321 N1	[3H]-Mesulergine	Mesulergine 1 μM	20 h / RT	SPA	hr 5HT2C	> 30
B	Muscarinic type 1 receptor assay , human	CHO-K1	[3H]-Scopolamine	Atropine 5 μM	20 h / RT	SPA	hr M1	> 30
F	Muscarinic type 2 receptor agonist assay	CHO-K1	n/a	Acetylcholine 3 μM	2 min / RT	Ca flux/ATP	hr M2 ag	> 30
F	Muscarinic type 2 receptor antagonist assay	CHO-K1	Acetylcholine 0.1 μM	AQ-RA741 5 μM	15 min / RT	Ca flux/ATP	hr M2 antag	> 30
B	Muscarinic type 3 receptor assay , human	CHO-K1	[3H]-Scopolamine	Atropine 5 μM	20 h / RT	SPA	hr M3	> 30
B	Melanocortin MC3 receptor binding assay,	HEK-293	[125I]-[NDP]alphaMSH	[NDP]alphaMSH 1 μM	10 h / RT	SPA	hr MC3	> 30
B	Motilin receptor binding assay : human	HEK-293	[125I]-Tyr7-Motilin	Motilin 1 μM	12 h / RT	SPA	hr Mot	> 30
B	Opiate Delta OpD receptor assay, human	HEK-293	[3H]-DADLE	Naloxone 50 μM	20 h / RT	SPA	hr OpD	> 30
B	Opiate Mu OpM receptor assay, human	CHO-K1	[3H]-Naloxone	Naloxone 20 μM	20 h / RT	SPA	hr OpM	29
F	Thromboxane A2 receptor (TP) agonist assay	HEK-293	n/a	U46619 1 μM	2 min / 37°C	Ca flux	hr TP ag	> 30
F	Thromboxane A2 receptor (TP) antagonist	HEK-293	U46619 75nM	ICI 192605 1 μM	30 min / 37°C	Ca flux	hr TP antag	9.4
B	Vasopressin V1a receptor assay : human	HEK-293	[125I]-lin.vasopressin antagonist	8-AVP 10 μM	20 h / RT	SPA	hr V1a	> 30
B	Adenosin Transporter assay, human	U937	[3H]-NBTI	NBTI 1 μM	10 h / RT	SPA	h AdT	> 30
F	Bile salt export pump (BSEP) vesicular	Insect Sf9	[3H]-Taurocholate	Cyclosporine A 50 μM	30 min / RT	Filtration	hr BSEP (n=2)	17
F	Vesicular monoamine transporter (VMAT2)	Rat brain cortex	[3H]-Dopamine	Reserpine 1 μM	40 min / RT	Filtration	rVMAT2uptake	0.33
B	Dopamine Transporter assay, human	CHO-K1	[3H]-WIN35428	GBR 12909 10 μM	7 h / RT	SPA	hr DAT	> 30
B	Norepinephrine Transporter assay, human	MDCK	[3H]-Nisoxetine	Nisoxetine 1 μM	10 h / RT	SPA	hr NET	> 30
B	Serotonin Transporter assay, human	HEK293	[3H]-Paroxetine	Imipramine 20 μM	20 h / RT	SPA	hr 5HTT	> 30
F	Adenosine Transporter assay, human	U937	[3H]-NBTI	NBTI 1 μM	10 h / RT	SPA	hr AdT	14
B	Benzodiazepine receptor assay, human	rat brain	[3H]-Flunitrazepam	Diazepam 10 μM	90 min / 4°C	Filtration	r BZD	> 30
F	GABA A, Benzodiazepine centrall agonist assay	CHO-K1	n/a	GABA 50 μM	15 min / RT	YFP	hr GABAA ag	> 30
F	GABA A, Benzodiazepine centrall antagonist	CHO-K1	GABA 1 μM	Bicuculline 50 μM	15 min / RT	YFP	hr GABAA antag	> 30
B	Nicotinic receptor assay, human recombinant	IMR32	[125I]-Epibatidine	(-)Nicotine 100 μM	15 h / RT	SPA	h Nic(cns)	> 30
B	Serotonin 5HT3 receptor assay, human	HEK293	[3H]-GR65630	MDL72222 10 μM	60 min / RT	SPA	hr 5HT3	> 30
F	Androgen receptor agonist assay	Insect	Coactivator = D11FxxLF (250nM)	Dihydrotestosterone 1 μM	1 h / RT	TR-FRET	rr AR ag	> 30
F	Estrogen receptor agonist assay	Insect	Coactivator = PGC1a (250nM)	17-beta Estradiol 1 μM	1 h / RT	TR-FRET	hr ERa ag	> 30
F	Estrogen receptor antagonist assay	Insect	17-beta Estradiol 7nM	4-OH Tamoxifen 1 μM	1 h / RT	TR-FRET	hr ERa antag	> 30
F	Farnesoid X receptor agonist assay	Insect	Coactivator = SRC2-2 (500 nM)	GW4064 10 μM	1 h / RT	TR-FRET	hr FXR ag	> 30
F	Farnesoid X receptor antagonist assay	Insect	GW4064 0.2 μM	Guggulsterone 100 μM	1 h / RT	TR-FRET	hr FXR antag	> 30
F	Glucocorticoid receptor agonist assay	Insect	Coactivator = SRC1-4 (250 nM)	Mometasone furoate 1 μM	1 h / RT	TR-FRET	hr GR ag	> 30
F	Glucocorticoid receptor antagonist assay	Insect	Mometasone furoate 23 nM	Mifepristone 1 μM	1 h / RT	TR-FRET	hr GR antag	> 30
F	Liver X receptors (LXR) alpha agonist assay	Insect	Coactivator = TRAP220/DRIP-2 (250 nM)	T 0901317 1 μM	1 h / RT	TR-FRET	hr LXRa ag	> 30
F	Liver X receptors (LXR) alpha antagonist assay	Insect	T 0901317 0.1 μM	Fenofibrate 100 μM	1 h / RT	TR-FRET	hr LXRa antag	> 30
F	Liver X receptors (LXR) beta agonist assay	Insect	Coactivator = D22 (100 nM)	T 0901317 10 μM	1 h / RT	TR-FRET	hr LXRb ag	> 30
F	Liver X receptors (LXR) beta antagonist assay	Insect	T 0901317 0.045 μM	Fenofibrate 100 μM	1 h / RT	TR-FRET	hr LXRb antag	> 30
F	Peroxisome proliferator-activated receptor alpha	Insect	Coactivator = PGC1 (250 nM)a	GW7647 1 μM	1 h / RT	TR-FRET	hr PPARa ag	> 30
F	Peroxisome proliferator-activated receptor alpha	Insect	GW7647 10 nM	GW9662 10 μM	1 h / RT	TR-FRET	hr PPARa antag	> 30
F	Peroxisome proliferator-activated receptor	Insect	Coactivator = TRAP220 (125nM)	Rosiglitazone 1 μM	1 h / RT	TR-FRET	hr PPARg ag (n=2)	> 30
F	Peroxisome proliferator-activated receptor	Insect	Rosiglitazone 50 nM	GW9662 10 μM	1 h / RT	TR-FRET	hr PPARg antag (n=2)	> 30
F	Progesterone receptor agonist assay	Insect	Coactivator = SRC1-4 (250nM)	Progesterone 100 nM	1 h / RT	TR-FRET	hr PR ag	> 30
F	Progesterone receptor antagonist assay	Insect	Progesterone 10 nM	Mifepristone 3 μM	1 h / RT	TR-FRET	hr PR antag	> 30
F	Pregnane X Receptor agonist assay	Insect	Coactivator = SRC1-4 (250nM)	SR12813 20 μM	1 h / RT	TR-FRET	hr PXR ag	> 30
F	Pregnane X Receptor antagonist assay	Insect	SR12813 0.8 μM	Zafirlukast 30 μM	1 h / RT	TR-FRET	hr PXR antag	> 30
E	Acetylcholinesterase asay, human recombinant	HEK-293	Acetylthiocholine iodide 0.35 mM	Physostigmine 10 μM	10 min / RT	Spectro-photometric	hr ACES	> 30
E	COX-1 assay, ovine recombinant	Isolated from ram seminal vesicles	Arachidonic Acid	Diclofenac 10 μM	30+30 min / RT	FI	or COX-1	> 30
E	COX-2 assay, human recombinant	Baculovirus transfected Sf21 cells	Arachidonic Acid	Diclofenac 10 μM	30+5 min / RT	FI	hr COX-2	> 30
E	Monoamine oxidase A assay, human	Baculovirus infected BTI-TN-5B1-4 cells	Luminogenic MAO-A substrate (a derivative of beetle luciferin)	Clorgyline 10 μM	2 h / RT	Luminescence	hr MAO A	> 30
E	Phosphodiesterase 3 from human platelets	h Platelet	[3H]-cAMP	Cilostamide 10 μM	30 min / RT	SPA	h PDE3	> 30
E	Phosphodiesterase 4D: human recombinant	baculovirus transfected-insect cells	[3H]-cAMP	Rolipram 5 μM	60 min / RT	SPA	hr PDE4D	> 30
E	EGFR kinase	Baculovirus transfected insect cells	fluorescently labeled peptide	Staurosporine 10 μM	1 h / RT	FI	hr EGFR	> 30
E	ERBB2 kinase	Baculovirus transfected insect cells	SRCtide 10 μM	Staurosporine 10 μM	30 min / RT	FI	hr ERBB2	> 30
E	KDR kinase	Baculovirus transfected insect cells	fluorescently labeled peptide	Staurosporine 10 μM	1 h / RT	FI	hr KDR	> 30
E	ACVR1 kinase	Baculovirus transfected insect cells	fluorescently labeled peptide	Staurosporine	1h / 30°C	Caliper	CACVR1 (172-R206H-499)	>10
E	AURKA kinase	Invitrogen/ThermoFisher Scientific	fluorescently labeled peptide	Staurosporine	1h / 30°C	Caliper	AURKA	>10
E	BTK kinase	Baculovirus transfected insect cells	fluorescently labeled peptide	Staurosporine	1h / 30°C	Caliper	BTK	>10
E	FGFR2 kinase	Baculovirus transfected insect cells	fluorescently labeled peptide	Staurosporine	1h / 30°C	Caliper	FGFR2 (406-821)	>10
E	FGFR4 kinase	Baculovirus transfected insect cells	fluorescently labeled peptide	Staurosporine	1h / 30°C	Caliper	FGFR4 (388-802)	>10
E	FLT3 kinase	Baculovirus transfected insect cells	fluorescently labeled peptide	Staurosporine	1h / 30°C	Caliper	FLT3 (563-D835Y-993)	>10
E	GSK3B kinase	Baculovirus transfected insect cells	fluorescently labeled peptide	Staurosporine	1h / 30°C	Caliper	GSK3B	>10
E	IRAK1 kinase	Baculovirus transfected insect cells	fluorescently labeled peptide	Staurosporine	1h / 30°C	Caliper	IRAK1 (184-712)	>10
E	IRAK4 kinase	Baculovirus transfected insect cells	fluorescently labeled peptide	Staurosporine	1h / 30°C	Caliper	IRAK4 (1-460)	>10
E	JAK2 kinase	Baculovirus transfected insect cells	fluorescently labeled peptide	Staurosporine	1h / 30°C	Caliper	JAK2 (808-1132)	>10
E	KDR kinase	Baculovirus transfected insect cells	fluorescently labeled peptide	Staurosporine	1h / 30°C	Caliper	KDR (807-1356)	>10
E	LYN kinase	Baculovirus transfected insect cells	fluorescently labeled peptide	Staurosporine	1h / 30°C	Caliper	LYN (1-512)	>10
E	MAP3K8 kinase	Baculovirus transfected insect cells	fluorescently labeled peptide	Staurosporine	1h / 30°C	Caliper	MAP3K8 (30-404)	>10
E	MAP4K4 kinase	Baculovirus transfected insect cells	fluorescently labeled peptide	Staurosporine	1h / 30°C	Caliper	MAP4K4	>10
E	PDGFRa kinase	Baculovirus transfected insect cells	fluorescently labeled peptide	Staurosporine	1h / 30°C	Caliper	PDGFRa (551-V561D-1089)	>10
E	PRKCA kinase	Invitrogen/ThermoFisher Scientific	fluorescently labeled peptide	Staurosporine	1h / 30°C	Caliper	PRKCA	>10
E	PRKCQ kinase	Invitrogen/ThermoFisher Scientific	fluorescently labeled peptide	Staurosporine	1h / 30°C	Caliper	PRKCQ	>10
E	ROCK2 kinase	ProQinase (Freiburg Germany)	fluorescently labeled peptide	Staurosporine	1h / 30°C	Caliper	ROCK2 (6-553)	>10
E	STK17B kinase	Baculovirus transfected insect cells	fluorescently labeled peptide	Staurosporine	1h / 30°C	Caliper	STK17B	>10
E	STK4 kinase	Invitrogen/ThermoFisher Scientific	fluorescently labeled peptide	Staurosporine	1h / 30°C	Caliper	STK4	>10
E	SYK kinase	Baculovirus transfected insect cells	fluorescently labeled peptide	Staurosporine	1h / 30°C	Caliper	SYK (2-635)	>10
E	ZAP70 kinase	Invitrogen/ThermoFisher Scientific	fluorescently labeled peptide	Staurosporine	1h / 30°C	Caliper	ZAP70	>10
E	ABL1 kinase	Baculovirus transfected insect cells	fluorescently labeled peptide	Staurosporine	1h / 30°C	Caliper	ABL1 (64-515) non-phos	>10
E	MAP3K7 kinase	Baculovirus transfected insect cells	fluorescently labeled peptide	Staurosporine	1h / 30°C	Caliper	MAP3K7 (1-303) - TAB1 (437-504)	>10
E	Beta-secretase 1	Baculovirus transfected insect cells	Ac-C(PT14)-E-V-N-L-D-A-E-W-K-NH2	specific reference inhibitor	1h / RT	FLT	hBACE1	>100
E	Beta-secretase 2	Baculovirus transfected insect cells	Ac-C(PT14)-E-V-N-L-D-A-E-W-K-NH2	specific reference inhibitor	1h / RT	FLT	hBACE2	>100
E	Caspase 3	E.coli	PT14-D-E-V-D-W-E-NH2	specific reference inhibitor	1h / RT	FLT	hCaspase3 FLT	>100
E	Cathepsin B	E. coli	Z-L-R-Rh110-γQ	specific reference inhibitor	1h / RT	FI	hCathepsinB	>100
E	Cathepsin C	Baculovirus transfected insect cells	Gly-R-Rh110-γQ	specific reference inhibitor	1h / RT	FI	hCathepsinC	>100
E	Cathepsin D	Baculovirus transfected insect cells	R-C(PT14)-K-P-I-L-F-F-R-L-G-W-R	specific reference inhibitor	1h / RT	FLT	s_hCathepsinD	>100
E	Cathepsin E	E.coli	R-C(PT14)-K-P-I-L-F-F-R-L-G-W-R	specific reference inhibitor	1h / RT	FLT	hCathepsinE	>100
E	Cathepsin G	Purified from neutrophils	Suc-A-A-P-F-Rh110-γQ	specific reference inhibitor	1h / RT	FI	hCathepsinG	>100
E	Cathepsin K	Baculovirus transfected insect cells	Z-L-R-Rh110-E	specific reference inhibitor	1h / RT	FI	hCathepsinK	>100
E	Cathepsin L	Baculovirus transfected insect cells	Z-F-R-Rh110-γQ	specific reference inhibitor	1h / RT	FI	hCathepsinL	>100
E	Cathepsin S	Baculovirus transfected insect cells	Z-L-R-Rh110-γQ	specific reference inhibitor	1h / RT	FI	hCathepsinS	>100
E	Cationic Trypsin	E.coli	Bz-G-P-R-Rh110-γQ	specific reference inhibitor	1h / RT	FI	hCatTrypsin	>100
E	Chymase	Baculovirus transfected insect cells	Suc-L-L-V-Y-AMC	specific reference inhibitor	1h / RT	FI	hChymase	>100
E	Chymotrypsin	purified from pancreas	Suc-A-A-P-F-Rh110-γQ	specific reference inhibitor	1h / RT	FI	hChymotrypsin	>100
E	Factor XIIa	purified from human plasma	D-P-F-R-Rh110-γQ	specific reference inhibitor	1h / RT	FI	hFactor XIIa	>100
E	Factor IXa	purified from human plasma	D-L-G-R-Rh110-dP-	specific reference inhibitor	1h / RT	FI	hFactorIXa	>100

E	FactorXa	purified from human plasma	Bz-I-E-G-R-Rh110-γQ	specific reference inhibitor	1h / RT	FI	hFactorXa	>100
E	Factor VIIa	purified from human plasma	D-F-P-R-Rh110-dP	specific reference inhibitor	1h / RT	FI	hFVIIa	>100
E	Kallikrein 1	Baculovirus transfected insect cells	Ac-G-W-S-P-F-R-S-S-L-C(PT14)-NH2	specific reference inhibitor	1h / RT	FLT	hKallikrein1	>100
E	Kallikrein 5	Recombinant, mammalian cell line	C(PT14)-F-R-W-NH2	specific reference inhibitor	1h / RT	FLT	hKallikrein5	>100
E	Kallikrein 7	E.coli	Ac-E-F-L-P-I-L-W-R-L-G-C(PT14)-E-NH2	specific reference inhibitor	1h / RT	FLT	hKallikrein7	79
E	Membrane type serine protease 1	E.coli	Ac-R-L-F-L-Rh110-γQ	specific reference inhibitor	1h / RT	FI	hMT-SP1	>100
E	Neutrophil Elastase	Leucocytes of Purulent Human Sputum	Ac-W-S-E-V-N-L-D-A-E-C(PT14)-NH2	specific reference inhibitor	1h / RT	FLT	HN Elastase	>100
E	Pancreatic elastase	purified from pancreas	Ac-W-S-E-V-N-L-D-A-E-C(PT14)-NH2	specific reference inhibitor	1h / RT	FLT	hPancELA1	>100
E	Plasmin	native	H-S-G-I-Y-L-S-S-C(PT14)	specific reference inhibitor	1h / RT	FLT	hPlasmin	>100
E	Plasma Kallikrein	purified from human plasma	D-P-F-R-Rh110-γQ	specific reference inhibitor	1h / RT	FI	hPlasmaKallikrein	>100
E	Proteinase 3	Leucocytes of Purulent Human Sputum	Ac-D-T-E-D-V-V-P-Nva-Rh110-γQ	specific reference inhibitor	1h / RT	FI	hProteinase3	>100
E	Renin	CHO cells	K-H-P-W-H-L-γV-I-H-T-C(PT14)-K	specific reference inhibitor	1h / RT	FLT	hRenin	>100
E	Thrombin	purified from human plasma	Bz-V-P-R-Rh110-dP-	specific reference inhibitor	1h / RT	FI	hThrombin	>100
E	Urokinase	Recombinant, mammalian cell line	H-Y-S-G-R-G-C(PT14)-NH2	specific reference inhibitor	1h / RT	FLT	hUrokinase	>100
E	Matrix metalloproteinase-8	E.coli	Ac-C(PT14)-K-P-L-G-L-W-A-R-NH2	specific reference inhibitor	1h / RT	FLT	MMP08	>100

Proteins in the table are of human origin unless annotated otherwise. For peptidic substrates, the 1-letter code to annotate amino acids is used, except Nva = Norvaline. PT14 = Puretime TM 14 (fluorescent dye), RH110 = Rhodamine 110, AMC = 7-Amino-4-Methylcoumarin, Ac = N-acetyl, Bz = N-benzoyl, Suc = N-succinyl

Supplementary Table 2. 4-week rat toxicity study: Mean toxicokinetic parameters for MLT-943 in rat plasma.

Day	Dose	T_{max}	C_{max}	C_{max} /Dose	$AUC_{0\text{-}24h}$	$AUC_{0\text{-}24h}$ /Dose	SE of $AUC_{0\text{-}24h}$	C_{trough}	AUC Interval
		Mean male MLT-943 toxicokinetic parameters							
1	5	1.0	2050	410	22900	4580	695	0	0-24
	20	8.0	6970	349	116000	5810	12800	0	0-24
	80	8.0	25200	315	466000	5830	13300	0	0-24
13	5	2.0	2110	422	24800	4950	887	179	0-24
	20	8.0	10400	520	179000	8940	9380	3160	0-24
	80	4.0	19600	245	310000	3870	62200	5590	0-24
28	5	1.0	1860	372	27000	5390	975	220	0-24
	20	8.0	11100	555	187000	9360	4060	2790	0-24
	80	4.0	22200	278	250000	3120	17800	2770	0-24
					Mean female MLT-943 toxicokinetic parameters				
1	5	4.0	1690	338	24500	4900	3030	0	0-24
	20	4.0	10000	500	146000	7310	10200	0	0-24
	80	8.0	23100	289	473000	5910	20500	0	0-24
13	5	1.0	3210	642	51400	10300	5170	1160	0-24
	20	1.0	16700	835	230000	11500	27100	4560	0-24
	80	4.0	26200	328	387000	4840	59600	6710	0-24
28	5	1.0	3790	758	60700	12100	3490	1530	0-24
	20	2.0	19200	960	273000	13600	21200	7230	0-24
	80	4.0	29100	364	387000	4840	73900	1300	0-24

T_{max} (h), C_{max} (ng/mL), C_{max}/Dose (ng/mL)/(mg/kg/day), $AUC_{0\text{-}24h}$ and SE of $AUC_{0\text{-}24h}$ (ng*Hours/mL), C_{trough} (ng/mL), AUC Interval (Hours), $AUC_{0\text{-}24h}$/Dose (ng*Hours/mL)/(mg/kg/day).

Supplementary Table 3. 13-week rat toxicity study: Mean MLT-943 toxicokinetic parameters in rat plasma**.**

Gender	Study	Dose	T_{max}	C_{max}	C_{max}/Dose	AUC	AUC/Dose	SE of Composite AUC (NJH)	C_{trough}
Male	1	5	4.00	1830	366	20900	4190	1160	0.00
		20	4.00	6720	336	110000	5500	13100	0.00
		80	8.00	32400	405	571000	7140	30500	0.00
	72	5	2.00	2870	574	32000	6390	2120	288
		20	2.00	11800	590	183000	9170	Not Calculable	4550
		80	2.00	21700	271	221000	2760	79400	2100
Female	1	5	1.00	2110	422	23900	4780	2650	0.00
		20	8.00	8210	411	144000	7180	14200	0.00
		80	4.00	27300	341	537000	6710	36100	0.00
	72	5	2.00	3940	788	69300	13900	2650	1220
		20	4.00	21500	1080	299000	14900	48200	4710
		80	8.00	30500	381	429000	5360	49600	1640

Units: Dose (mg/kg/day); T_{max} (h); C_{max} (ng/mL); C_{max}/dose (ng/mL)/(mg/kg/day); AUC (ng*h/mL); AUC /dose (ng*h/mL) / mg/kg/day), C_{trough} (ng/mL), AUC interval (0-24h) and SE of Composite AUC (NJH, Nedelman-Jia-Holder Method) (ng*hours/mL).

Supplementary Table 4. 4-week dog toxicity study: Mean toxicokinetic parameters for MLT-943 in dog plasma.

Day	Dose	T_{max}	C_{max}	CV% of C_{max}	C_{max} /Dose	AUC_{0-24h}	CV% of AUC_{0-24h}	AUC_{0-24h} /Dose	C_{trough}	AUC Interval	n
		Mean male MLT-943 toxicokinetic parameters									
1	0.5	1.0	339	10.9	678	5340	6.4	10700	0	0-24	3
	2	1.33	1350	16.7	673	20400	14.2	10200	0	0-24	3
	5	3.2	2800	22.1	559	52500	20.0	10500	0	0-24	5
11	0.5	1.0	736	21.5	1470	12000	25.7	24000	369	0-24	3
	2	1.33	2710	27.6	1360	45500	29.7	22800	1430	0-24	3
	5	1.8	10700	37.3	2150	227000	36.1	45400	8510	0-24	5
24	0.5	1.0	716	19.7	1430	11600	28.8	23300	376	0-24	3
	2	2.0	2580	28.4	1290	45700	26.0	22800	1480	0-24	3
	5	3.6	9620	34.8	1920	204000	37.7	40800	7580	0-24	5
		Mean female MLT-943 toxicokinetic parameters									
1	0.5	1.0	368	9.78	737	5780	8.3	11600	0	0-24	3
	2	3.0	1300	23.2	652	23200	25.0	11600	0	0-24	3
	5	4.0	2730	13.5	545	55200	9.8	11000	0	0-24	5
11	0.5	1.33	779	8.32	1560	12800	7.09	25700	388	0-24	3
	2	2.0	3740	44.7	1880	73200	47.7	36600	2740	0-24	3
	5	3.6	12200	25.8	2440	268000	28.4	53600	10200	0-24	5
24	0.5	1.0	800	5.53	1600	12700	4.48	25500	419	0-24	3
	2	1.33	4440	45.0	2220	90500	55.1	45300	3380	0-24	3
	5	5.2	13300	36.5	2660	302000	40.4	60400	11900	0-24	5

Units: T_{max} (h), C_{max} (ng/mL), C_{max}/Dose (ng/mL)/(mg/kg/day), AUC_{0-24h} (ng*Hours/mL), C_{trough} (ng/mL), AUC_{0-24h}/Dose (ng*Hours/mL)/(mg/kg/day), AUC Interval (Hours).

Supplementary Table 5. 13-week dog toxicity study: Mean plasma MLT-943 toxicokinetic parameters.

Gender	Study Day	Dose	n	T_{max}	SD	C_{max}	SD	C_{max} /Dose	SD	AUC	SD	AUC /Dose	SD	C_{trough}	SD
Male	1	2	3	1.33	0.577	1560	130	780	65.0	31100	4110	15600	2050	0.00	0.00
		5	3	1.33	0.577	2660	693	533	139	51800	10900	10400	2180	0.00	0.00
		10	5	4.40	2.51	5320	1130	532	113	115000	28300	11500	2830	0.00	0.00
	55	7	5	2.60	1.95	31000	11300	4430	1620	688000	267000	98300	38400		
	90	2	3	1.00	0.00	5120	2150	2570	1080	99700	46100	49900	23100	3750	1780
		5	3	1.00	1.00	14600	4900	2920	977	323000	118000	64600	23600	13000	5570
		7	4	1.50	1.73	25300	10300	3610	1480	534000	272000	76400	39100	21200	12800
Female	1	2	3	1.00	0.00	1370	469	684	234	22900	7090	11400	3530	0.00	0.00
		5	3	3.33	3.21	2670	738	534	148	56000	16100	11200	3190	0.00	0.00
		10	5	3.20	1.10	5240	1190	524	119	110000	24100	11000	2410	0.00	0.00
	55	7	4	9.00	10.3	39700	8330	5670	1190	885000	187000	126000	26800		
	90	2	3	1.00	0.00	3530	1280	1770	645	64200	23200	32100	11600	2210	802
		5	3	1.67	0.577	11800	4790	2360	957	258000	116000	51500	23300	9600	4840
		7	4	4.75	1.50	22600	4660	3230	667	474000	95400	67700	13600	17600	3920

Units: Dose (mg/kg/day); T_{max} (h); C_{max} (ng/mL); C_{max}/dose (ng/mL) / (mg/kg/day); AUC (ng*hs/mL); AUC/dose (ng*h/mL) / mg/kg/day), C_{trough} (ng/mL) and AUC interval (0-24h).
