# Supplementary material for: Pharmacological Inhibition of MALT1 Protease Leads to a Progressive IPEX-Like Pathology
Source: Front Immunol. 2020 Apr 30;11:745. doi: 10.3389/fimmu.2020.00745 (PMC7203682; doi:10.3389/fimmu.2020.00745)
Supplement: Supplementary file 2 [file Data_Sheet_2.pdf]

Supplementary Figure 1

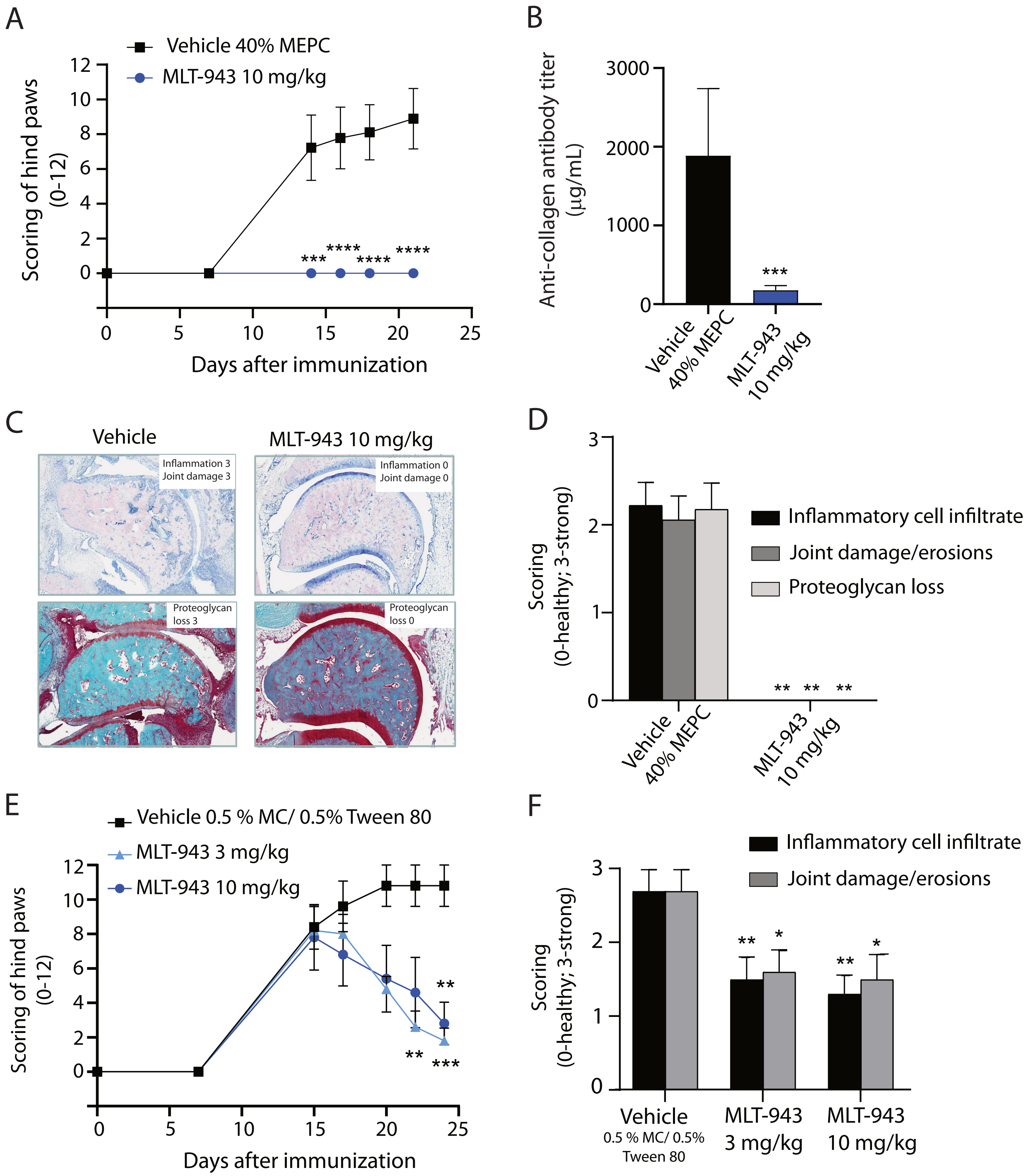

Supplementary Figure 1: Rats were immunized on day 0 and boosted on day 7 with collagen in IFA. **A)-D)** Compound was given p.o. daily from day 0 to day 20. **A)** Graphs showing hind paw swelling (mean± SEM) of rats during collagen-induced arthritis. **B)** Rat anti-collagen antibodies were measured in serum on day 21 (mean ± SEM). **C)** Histo-pathological scoring of hind paws based on staining with Giemsa for detection of inflammatory cell infiltrates and bone erosion, and with safranin O for proteoglycan loss indicating cartilage damage. **D)** Quantification of histological scoring. **E)-F)** Compound was given p.o. daily from day 15 to day 23. **E)** Graphs showing hind paw swelling (mean± SEM). **F)** Histo-pathological scoring of hind paws as in D). Statistics: 2-way ANOVA. * $p<0.05$, ** $p<0.01$, ***$p<0.001$, ****$p<0.0001$.

Supplementary Figure 2

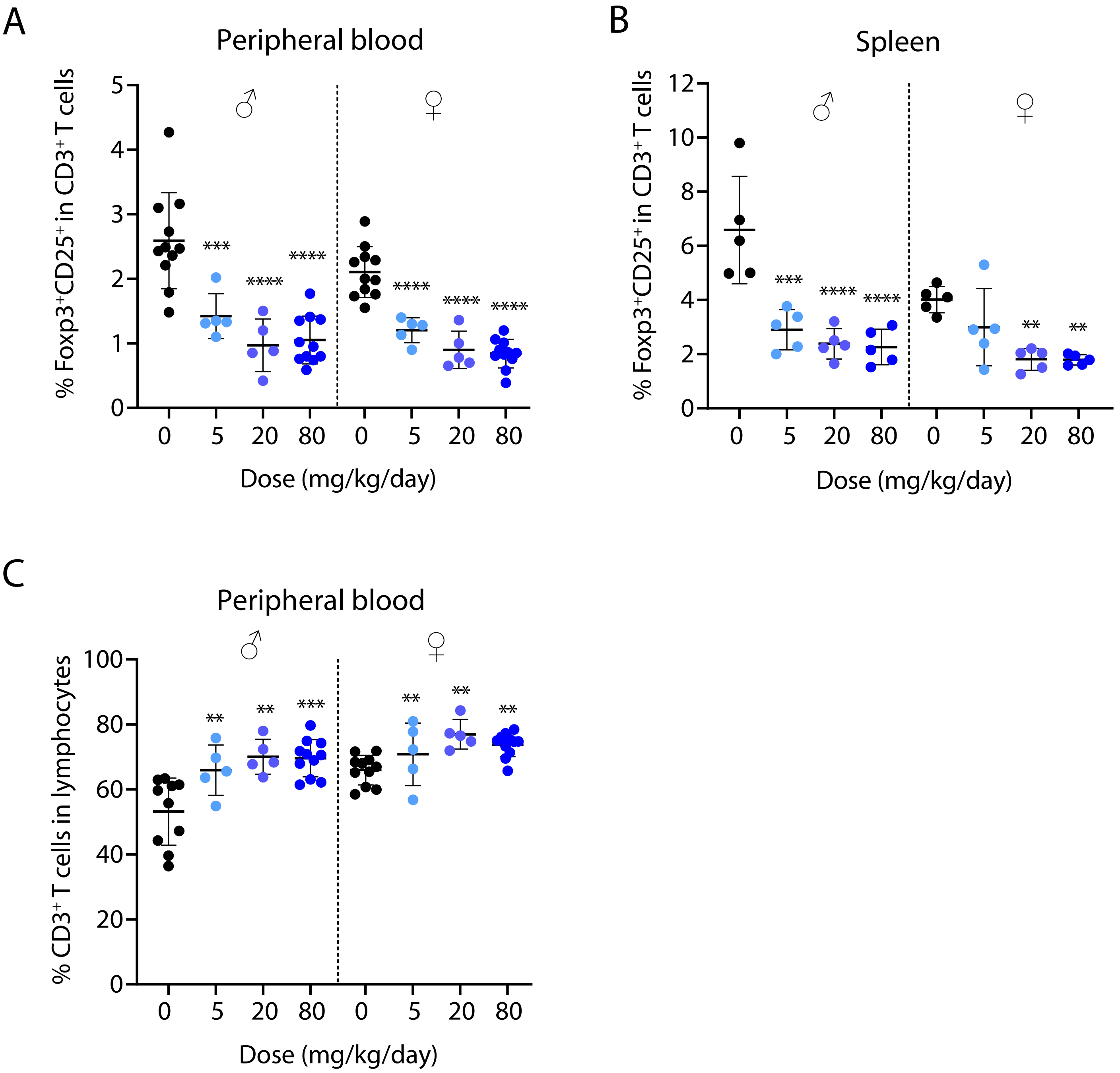

Supplementary Figure 2. Four weeks pharmacological MALT1 protease inhibition leads to reduced Tregs and increased T cell proportions in rats. **A+B)** Frequency of Foxp3$^+$CD25$^+$ Tregs in **A)** blood and **B)** spleen in male and female rats determined by FACS at time of necropsy (day 25). **C)** Frequency of CD3$^+$ T cells in blood at time of necropsy (day 25). Each dot represents an individual animal. Lines depict mean ± SD. Statistical difference was determined using one-way ANOVA with follow up for significance by multiple comparison tests with Sidak's correction, * $p< 0.05$, ** $p < 0.01$, *** $p < 0.001$, **** $p < 0.0001$.

Supplementary Figure 3

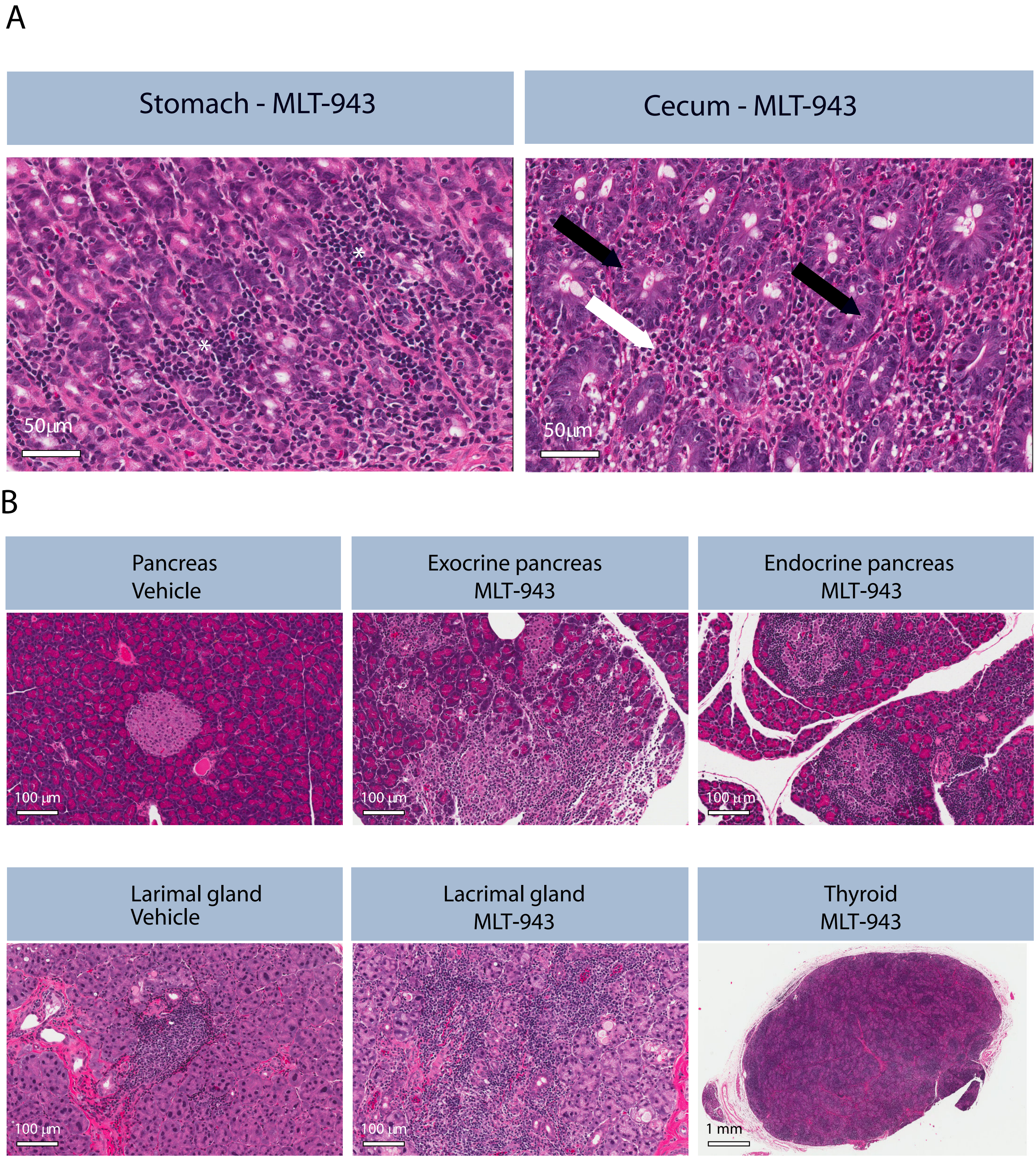

Supplementary Figure 3 Immune-mediated pathology is induced upon prolonged treatment with MLT-943. Histological alterations in Wistar rats treated with MLT-943 for up to 13 weeks. Sections shown are from both male and female animals of the vehicle or 80 mg/kg/day groups, except for the lacrimal gland MLT-943 section, which was taken from an animal of the 20 mg/kg/day group. Sections taken at necropsy day 86 (week 13) and stained with H&E. **A)** Enlarged version of pictures shown in Figure 3C: MLT-943, 13 week treatment samples in stomach and cecum. Mononuclear cell infiltrates (*), mixed cell infiltrates (white arrow), globular hyalin bodies (black arrows). **B)** Mononuclear cell infiltration in pancreas, lacrimal glands and the thyroid.

Supplementary Figure 4

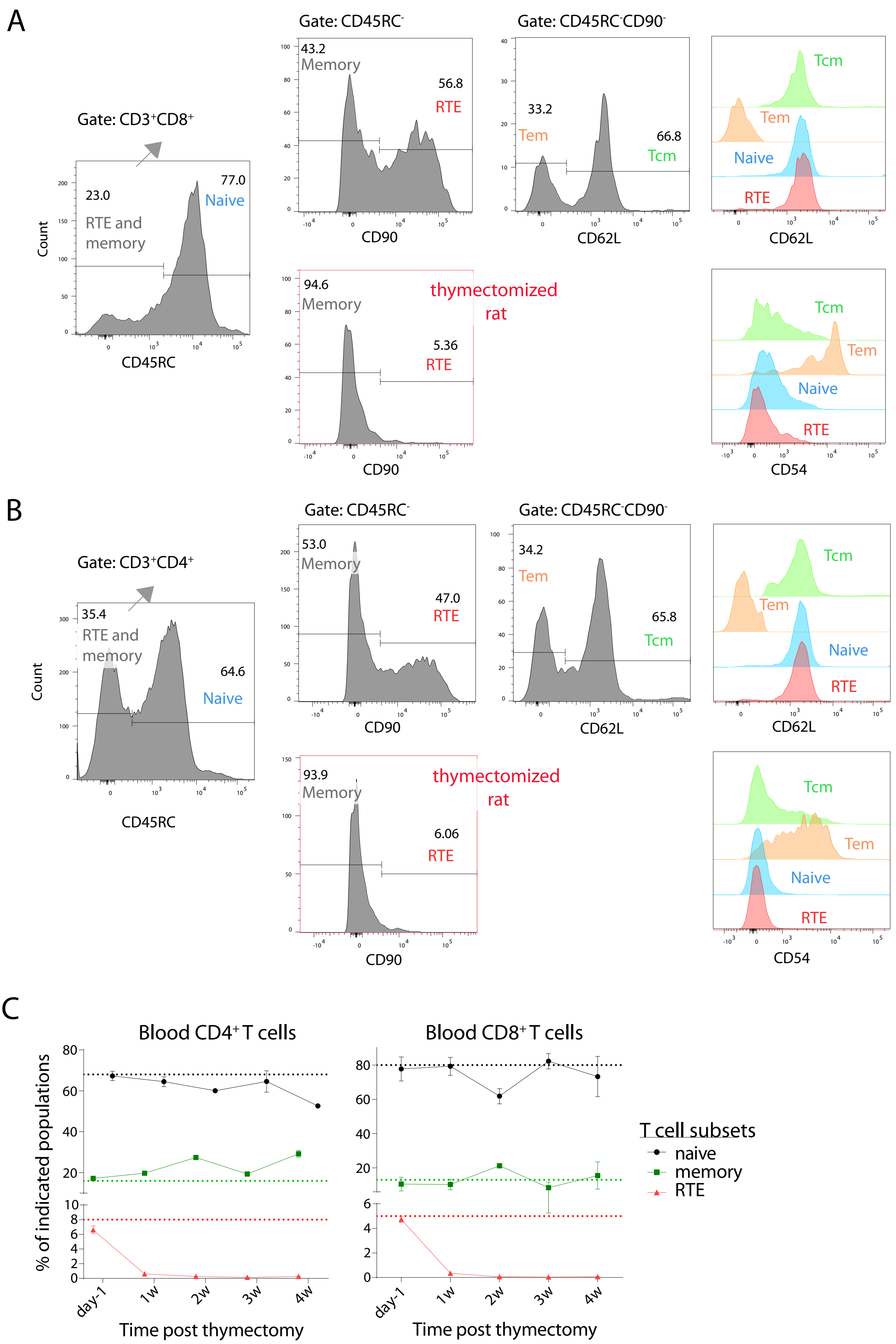

Supplementary Figure 4. Gating strategy for identification of recent thymic emigrants (RTEs), naive and memory T cell subsets in rats. **A)** $CD3^+CD8^+$ or **B)** $CD3^+CD4^+$ T cells were gated on CD45RC to discriminate $CD45RC^-$ RTEs and memory T cells from $CD45RC^+$ naive T cells. $CD45RC^-$ cells were further subdivided into $CD90^+$ RTEs and $CD90^-$ memory subsets. Finally, $CD45RC^-$ $CD90^-$ memory T cells can be divided into central memory and effector memory based on expression of CD62L (staining example on an euthymic rat). The red-framed plots shows the lack of RTEs in a thymectomized rat. **C)** Kinetic of different T cell subsets in thymectomized ratsfor $CD4^+$ T cells (left) and $CD8^+$ T cells (right). Points depict mean ± SEM.

Supplementary Figure 5

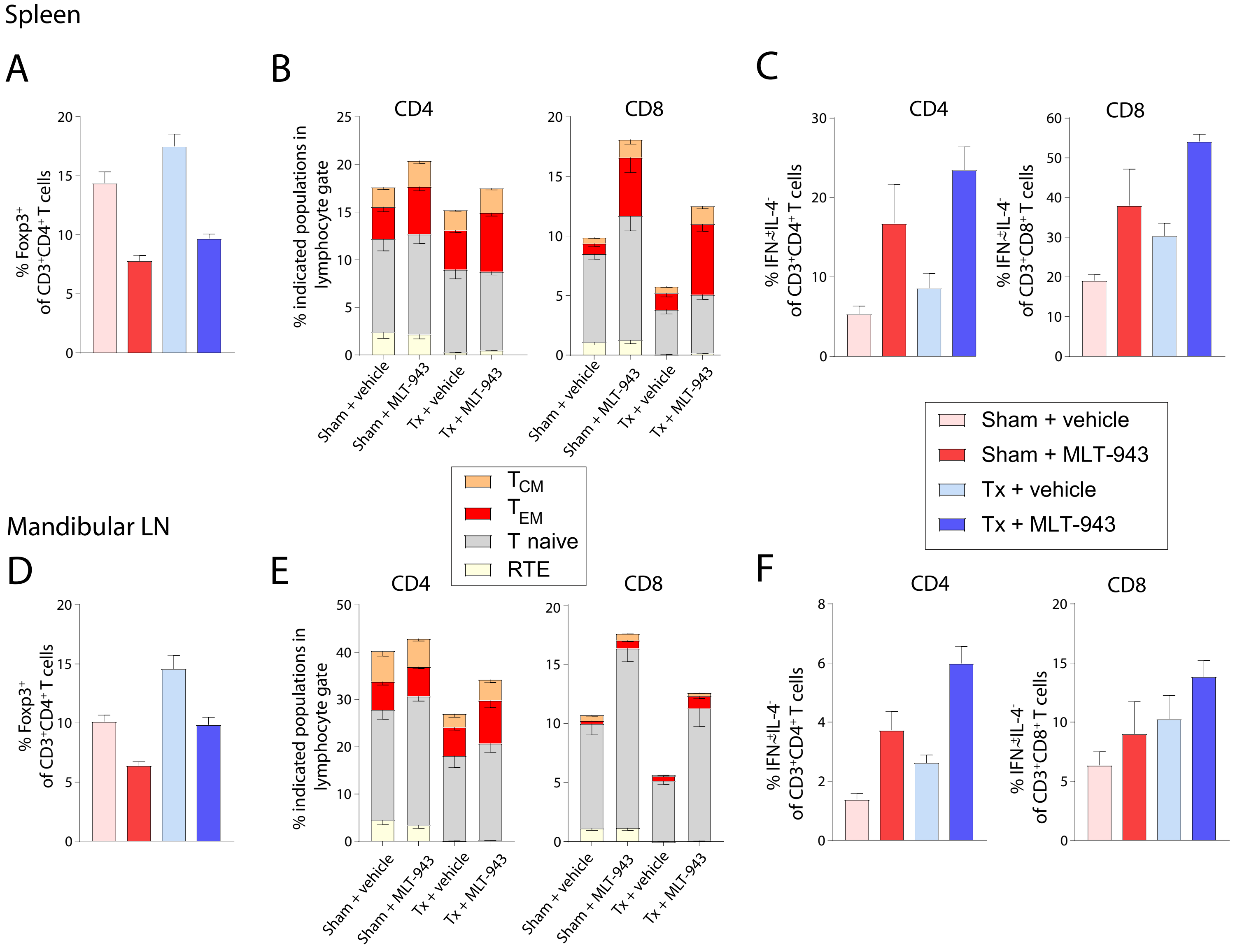

Supplementary Figure 5. Effects of MLT-943 treatment on T cell subsets in euthymic versus thymectomized rats. **A-F)** Spleens and mandibular LNs from male and female rats were harvested at necropsy (day 63) and analyzed by FACS. **A-C)** Frequency of **A)** Foxp3+CD25+ Tregs and **B)** indicated T cell subpopulations in spleen in the indicated groups. **C)** Percentage of IFNγ-producing, IL-4-negative CD4 (left graph) and CD8 (right graph) T cells isolated from spleen, detected by intracellular FACS staining after 4 hours PMA/Ionomycin stimulation ex vivo. **D-F)** T cell populations as described for A-C) isolated from mandibular LNs. Graphs depict means ± SEM.

Supplementary Figure 6

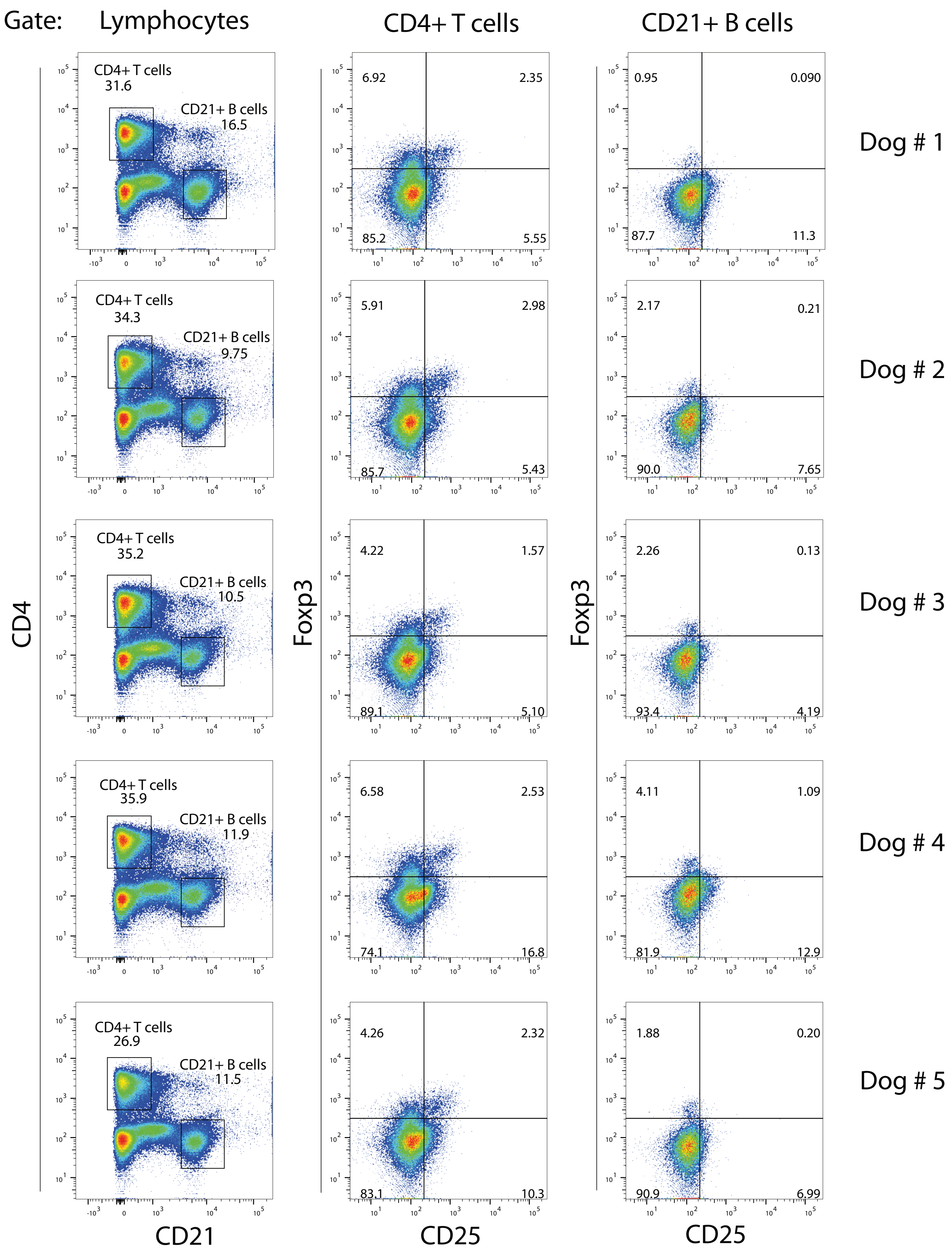

Supplementary Figure 6. Gating for Foxp3$^+$CD25$^+$ Tregs within CD4$^+$ T cells in dogs as exemplified in 5 naive dogs. Lymphocytes were seperated in CD4$^+$ T cells and CD21$^+$ B cells (left panels). CD4$^+$ T cells were further analyzed for Foxp3$^+$CD25$^+$ double-positive Tregs (middle panels). Foxp3 expression on CD21$^+$ B cells was assessed as negative staining control (right panels).

Supplementary Figure 7

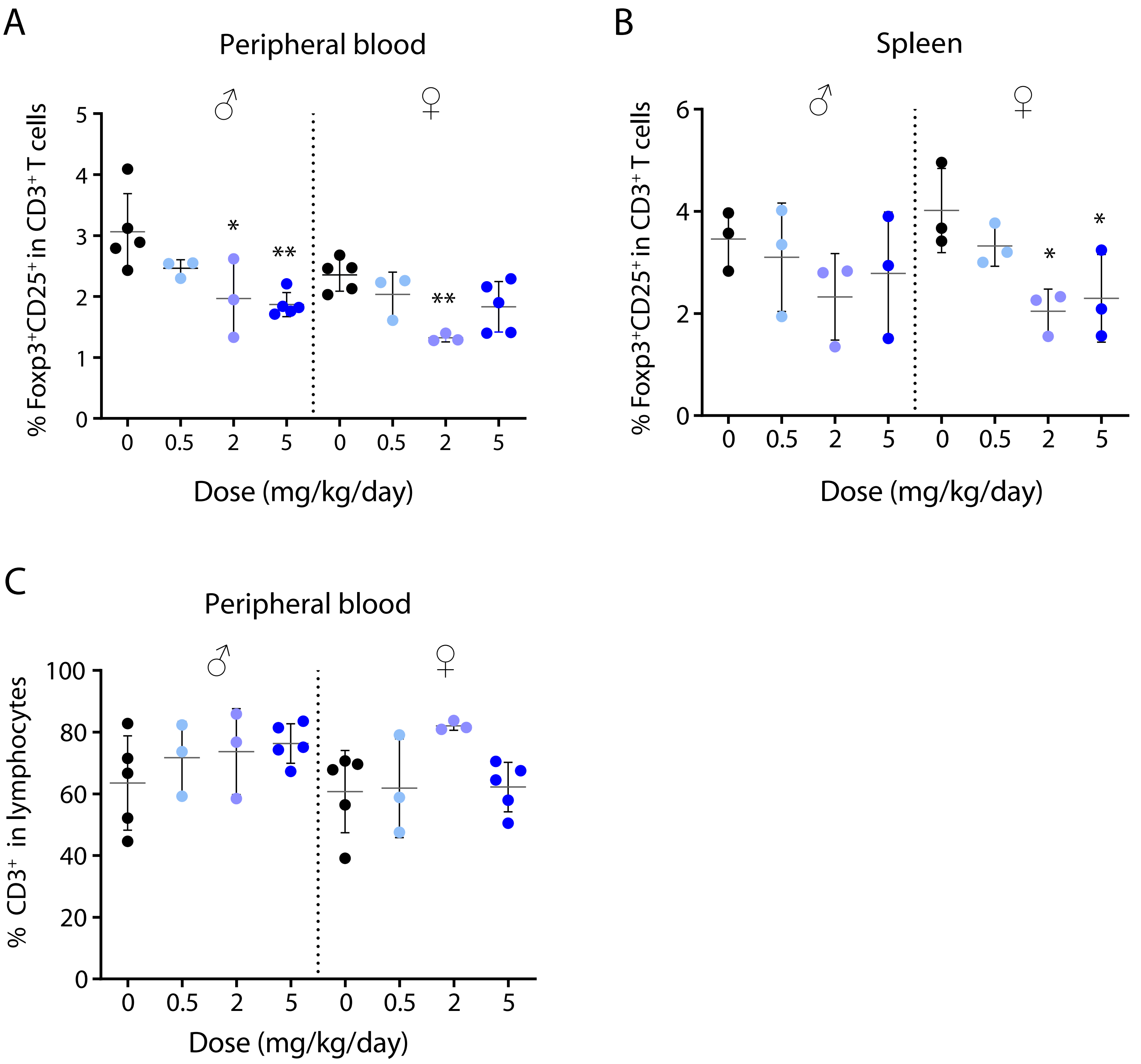

Supplementary Figure 7. Four weeks pharmacological MALT1 protease inhibition leads to reduced Treg proportions in dogs. **A+B)** Frequency of $Foxp3^{+}CD25^{+}$ Tregs in **A)** blood and **B)** spleen in male and female dogs determined by FACS at time of necropsy (day 25). **C)** Frequency of $CD3^{+}$ T cells in blood at time of necropsy (day 25). Each dot represents an individual animal. Lines depict mean ± SD. Statistical difference was determined using one-way ANOVA with follow up for significance by multiple comparison tests with Sidak's correction, * $p < 0.05$, ** $p < 0.01$, *** $p < 0.001$, **** $p < 0.0001$.
