# Supplementary material for: Pharmacological Inhibition of MALT1 Protease Leads to a Progressive IPEX-Like Pathology
Source: Front Immunol. 2020 Apr 30;11:745. doi: 10.3389/fimmu.2020.00745 (PMC7203682; doi:10.3389/fimmu.2020.00745)
Supplement: Supplementary file 3 [file Data_Sheet_3.pdf]

Synthesis of MLT-943

General information. All reagents were purchased from commercial sources and used without further purification. ^{1}H- and ^{13}C-NMR spectra were measured on various Bruker spectrometers at room temperature and chemical shifts are reported in parts per million (ppm) relative to an internal solvent reference. Peaks are tabulated in the order multiplicity (s, singlet; d, doublet; t, triplet; q, quartet; quintet; m, multiplet; br, broad), coupling constant and number of protons.

Purity and mass data was determined by analytical UPLC/MS (Waters) using the following method: Waters UPLC; column: Acquity HSS T3 1.8 µm, 2.1*50 mm, at 60°C, Eluent A: H2O + 0.05 % HCOOH + 3.75 mM ammonium acetate, B: CH3CN + 0.04 % HCOOH, Gradient: 10 to 95 % B in 1.5 min, Flow: 1 ml/min. Mass spectra over a mass range from 100 m/z to 1200 m/z are acquired using Electrospray Ionization (ESI) with positive/negative ion-switching.

High Resolution Mass Spectrometry analyses were performed by using electrospray ionization in positive ion modus after separation by liquid chromatography. The elemental composition was derived from the mass spectra acquired at the high resolution of about 35,000 on an Q Exactive Plus mass spectrometer (Thermo Scientific) coupled to an Ultimate 3000 UHPLC (Thermo Scientific). The high mass accuracy below 1.5 ppm was obtained by using a lock mass.

Synthesis scheme:

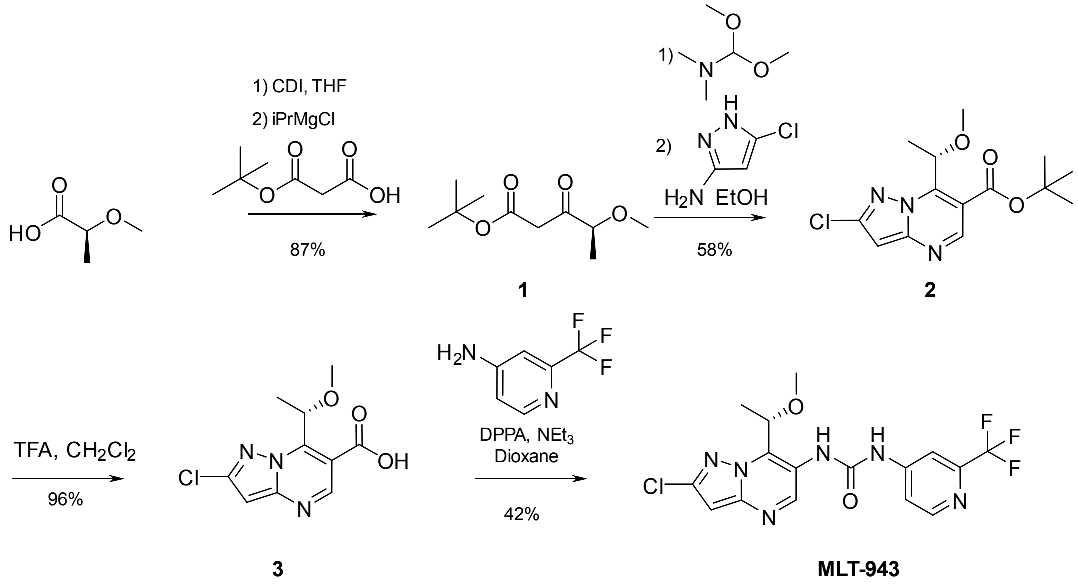

(S)-tert-butyl 4-methoxy-3-oxopentanoate 1: To a solution of (S)-2-methoxypropanoic acid (9.0 g, 96 mmol) in THF (170 mL) at 0 °C was added CDI (15.42 g, 95 mmol) and the reaction mixture was stirred at

this temperature for 1.25 h. In a separate flask, to a solution of 3-(tert-butoxy)-3-oxopropanoic acid (22.2 mL, 144 mmol) in THF (170 mL) at 0 °C was added dropwise 2 M isopropylmagnesium chloride in THF (125 mL, 251 mmol) and the reaction mixture was stirred for 1.25 h at 20 °C. Then, this solution was added dropwise to the acyl imidazole solution at 0 °C and the resulting mixture was stirred overnight at RT. The reaction mixture was quenched with 10% aqueous citric acid (25 mL), extracted with AcOEt, washed with aqueous saturated $NaHCO_3$, dried over Na_2SO_4, filtered and concentrated. The residue was purified by flash column chromatography on silica gel (cyclohexane/AcOEt: 100/0 to 80/20) to afford (S)-tert-butyl 4-methoxy-3-oxopentanoate **1** (16.1 g, 87%). M/z = 203 [M+H]+, Rt = 0.91 min, ^{1}H NMR (400 MHz, DMSO-d_6) δ ppm: 3.85 (q, *J* = 6.8 Hz, 1H), 3.54-3.46 (m, 2H), 3.27 (s, 3H), 1.40 (s, 9H), 1.19 (d, *J* = 6.8 Hz, 3H).

(S)-tert-butyl 2-chloro-7-(1-methoxyethyl)pyrazolo[1,5-a]pyrimidine-6-carboxylate 2: A mixture of 1,1-dimethoxy-N,N-dimethylmethanamine (7.6 g, 63.8 mmol) and (S)-tert-butyl 4-methoxy-3-oxopentanoate **1** (12.9 g, 94 mmol) was stirred at 120 °C for 1.5 h. Then a solution of 5-chloro-1H-pyrazol-3-amine (11.0 g, 94 mmol) in EtOH (100 mL) was added and the resulting mixture was stirred 1 h at 85 °C. The reaction mixture was concentrated and the residue was purified by flash column chromatography on silica gel (cyclohexane/AcOEt: 100/0 to 70/30). Enantiomeric excess (ee) was measured as 91% and the compound was further purified by chiral separation using a Thar SFC 200 instrument with a Chiralpak IC 5 µm column (30 × 250 mm), isocratic conditions (CO_2/isopropanol 95:5) to provide (S)-tert-butyl 2-chloro-7-(1-methoxyethyl)pyrazolo[1,5-a]pyrimidine-6-carboxylate **2** with ee > 99% (11.57 g, 58%). Enantiomeric purity was determined using a Waters UPC2 instrument with a Chiralpak IC 5 µm column (4.6 × 250 mm), isocratic conditions (CO_2/acetonitrile 85:15). M/z = 312 [M+H]+, Rt = 1.11 min, ^{1}H NMR (400 MHz, DMSO-d_6) δ ppm: 8.65 (s, 1H), 7.03 (s, 1H), 5.26 (q, *J* = 6.6 Hz, 1H), 3.22 (s, 3H), 1.62 (d, *J* = 6.6 Hz, 3H), 1.55 (s, 9H).

(S)-2-chloro-7-(1-methoxyethyl)pyrazolo[1,5-a]pyrimidine-6-carboxylic acid 3: To a solution of (S)-tert-butyl 2-chloro-7-(1-methoxyethyl)pyrazolo[1,5-a]pyrimidine-6-carboxylate **2** (10.37 g, 33.3 mmol) in DCM (50 mL) at RT was added TFA (61 mL). The reaction mixture was stirred overnight and concentrated. Et_2O was added to the residue was evaporated to dryness to afford (S)-2-chloro-7-(1-methoxyethyl)pyrazolo[1,5-a]pyrimidine-6-carboxylic acid **3** (8.85 g, 96%). M/z = 256 [M+H]+, Rt = 0.57 min, ^{1}H NMR (400 MHz, DMSO-d_6) δ ppm: 8.72 (s, 1H), 7.03 (s, 1H), 5.40 (q, *J* = 6.7 Hz, 1H), 3.20 (s, 3H), 1.64 (d, *J* = 6.7 Hz, 3H).

(S)-1-(2-chloro-7-(1-methoxyethyl)pyrazolo[1,5-a]pyrimidin-6-yl)-3-(2-(trifluoromethyl)pyridin-4-yl)urea MLT-943: To a solution of (S)-2-chloro-7-(1-methoxyethyl)pyrazolo[1,5-a]pyrimidine-6-carboxylic acid **3** (1.3 g, 4.78 mmol) in 1,4-dioxane (10 ml) were added DPPA (1.24 ml, 5.74 mmol) and Et_3N (3.33 ml, 23.9 mmol). The reaction mixture was stirred at RT for 30 min. Then, 2-(trifluoromethyl)pyridin-4-amine (1.55 g, 9.56 mmol) was added and reaction mixture was stirred at 100°C for 2h. The mixture was partitioned between AcOEt and saturated aqueous $NaHCO_3$ and the phases were separated. The organic layer was dried over Na_2SO_4, filtered and concentrated. The crude material was purified by flash column chromatography on silica gel (cyclohexane/AcOEt: 1/0 to 0/1). The residue was then taken up in MeOH

and heated until disolution. After cooling to RT the precipitate was collected by filtration, washed with MeOH and dried to afford (S)-1-(2-chloro-7-(1-methoxyethyl)pyrazolo[1,5-a]pyrimidin-6-yl)-3-(2-(trifluoromethyl)pyridin-4-yl)urea **MLT-943** (0.84 g, 42%). M/z = 415 [M+H]+, Rt = 1.12 min , ^{1}H NMR (400 MHz, DMSO-d6), δ: 10.36 (s, 1H), 8.92 (s, 1H), 8.56 (d, *J=5.6 Hz*, 1H), 8.06 (d, *J=2.0 Hz*, 1H), 7.61 (dd, *J=5.6, 2.0 Hz*, 1H), 6.92 (s, 1H), 5.41 (q, *J=6.7 Hz*, 1H), 3.32 (s, 3H), 1.57 (d, *J=6.7 Hz*, 3H). ^{13}C NMR (101 MHz, DMSO-d6) δ: 152.79, 150.85, 149.97, 148.03, 147.3 (q), 146.35, 144.45, 138.88, 121.6 (q), 120.03, 114.86, 108.89, 95.47, 72.93, 57.24, 17.40. HR-MS: $[M+H]^+$ C16H15ClF3N6O2 calc: 415.08916, found: 415.08914.
